# From meat to raw material: the Middle Pleistocene elephant butchery site of Casal Lumbroso (Rome, central Italy)

**DOI:** 10.1371/journal.pone.0328840

**Published:** 2025-10-08

**Authors:** Beniamino Mecozzi, Ivana Fiore, Biagio Giaccio, Francesca Giustini, Stefano Mercurio, Lorenzo Monaco, Alessia Argento, Francesco Bucci Casari degli Atti di Sassoferrato, Isabella Caricola, Cristina Lemorini, Francesco Lucchini, Ilaria Mazzini, Maria Rita Palombo, Raffaele Sardella, Andrea Sposato, Enza Elena Spinapolice, Francesca Alhaique

**Affiliations:** 1 Dipartimento di Biologia Ambientale, Sapienza Università Di Roma, Rome, Italy; 2 Dipartimento di Scienze Della Terra, Sapienza Università di Roma, Rome, Italy; 3 Servizio di Bioarcheologia, Museo delle Civiltà, Rome, Italy; 4 Consiglio Nazionale Delle Ricerche (CNR), Istituto di Geologia Ambientale e Geoingegneria, Area della Ricerca di Roma 1, Rome, Italy; 5 Dipartimento di Scienze delle Antichità, Sapienza Università di Roma, Rome, Italy; 6 Consiglio Nazionale delle Ricerche (CNR), Istituto di Scienze Marine, Area della Ricerca di Bologna, Bologna, Italy; 7 Soprintendenza Speciale Archeologia Belle Arti e Paesaggio di Roma, Rome, Italy; 8 School of Archaeology and Maritime Cultures, Haifa University, Haifa, Israel; Tel Aviv university, ISRAEL

## Abstract

The site of Casal Lumbroso is located in the north-west sector of Rome (central Italy). Stratigraphic and geochemical data presented here evidence that the archaeological and paleontological horizon lies at the top of the Tiber River aggradational succession related to the MIS 11c sea level highstand (dated at ca. 404 ka), and that the paleohabitat was characterised by wooded environments and humid climatic conditions. Paleontological analysis allows attributing most of the remains to an adult individual of straight-tusked elephant, *Palaeoloxodon antiquus*, with sporadic elements referred to *Stephanorhinus* sp., Bovinae, Cervinae, *Cervus elaphus*, *Dama* sp., *Canis* sp., *Oryctolagus* sp., *Talpa* sp., Testudines, and Amphibia. Two bird remains are referred to Anatidae and Strigiformes. A rich lithic assemblage, mainly made of flint, was also found associated with the fossil remains. Taphonomic, technological and functional analyses indicate that the *P. antiquus* carcass was probably exploited by humans not only as a food source, but also as a source of raw material, as documented by the presence of several intentionally fractured elephant bone fragments, some of them also with flake removals, with localized use wear traces. The findings at Casal Lumbroso highlight once again the importance of the territory around the city of Rome for Middle Pleistocene studies. The northwestern sector of the city, where other important sites such as Castel di Guido and La Polledrara di Cecanibbio have also been discovered, is therefore crucial for understanding human strategies for exploiting elephant carcasses.

## Introduction

The elephant-hominin interaction is documented in less than twenty localities across Europe, where fossils of *Palaeoloxodon antiquus* display clear evidence of human exploitation [1–3]. One of the main pieces of evidence for such interaction, often referred to as elephant butchery sites, is the exploitation of elephant carcasses as a source of meat, fat, and marrow, with bones fractured and showing cut marks [4–7]. However, the occurrence of cut marks on elephant bones is quite rare, and their presence or absence may depend on various factors involved in the butchery process [8–10]. Moreover, cut marks on elephant remains may be underrepresented because their thick and massive bones make it easier for butchery activities to be carried out without leaving visible traces. Proboscideans may indeed guarantee a significant amount of fat and meat, thereby serving as an important source of energy for human groups [11–14]. At many of these sites, elephant bones have also been used as raw material to produce various types of artifacts, especially when the local lithic raw material is not suitable for large tool production [4–7,15,16]. In the first place, the production of large tools would have been necessary, particularly due to their potential role in butchery, which would have required early humans to produce additional large tools during or after the butchery process [4–7,15,16]. Renowned European examples of elephant butchery sites are Marathousa 1 in Greece [4], Schöningen and Bilzingsleben in Germany [17–18], Áridos 1,2 in Spain [19], and Castel di Guido and La Polledrara di Cecanibbio in Italy [3,12].

However, methodological approaches introduced in recent decades have highlighted the need to consider multiple factors when interpreting an accumulation of lithic tools and elephant carcasses as a butchery event, rather than relying solely on the mere spatial association [2,8].

In south-western Eurasia, the palaeo-delta environment of the Tiber River, in central Italy, stands out as one of the Middle Pleistocene areas richest in large mammal remains and Lower Palaeolithic archaeological sites (see Supplementary Information), thus an ideal setting for investigating the interaction between hominins and other large mammals, including elephants, during the major climatic and environmental change occurring during the Early-Middle Pleistocene transition (EMPT), and especially after the Mid-Brunhes Event (MBE, ca. 424 ka; Marine Isotope Stages [MIS] 12–11 transition). The discovery of the archaeological and palaeontological horizon at Casal Lumbroso confirms the extraordinary importance of the territory around Rome, the so-called “Campagna Romana” for examining the impact of climatic changes on terrestrial ecosystems and human occupation during the Middle Pleistocene. At Casal Lumbroso, faunal remains include mainly a single carcass of *Palaeoloxodon antiquus* with relatively few other specimens belonging to other taxa, discovered alongside lithic artifacts and bone tools crafted from elephant bones. Casal Lumbroso thus appears particularly relevant for the study of human-elephant interactions, since in the same area the almost coeval sites of Castel di Guido and La Polledrara di Cecanibbio [3,12] have already been identified and studied. In this paper we present new preliminary data on the elephant butchery site of Casal Lumbroso using evidence from extensive archaeological and palaeontological excavation with the aim of improving our knowledge about terrestrial ecosystems and subsistence strategies of pre-Neanderthal populations. Based on stratigraphic and tephrochronological data, Casal Lumbroso is attributed to the MIS 11c, acknowledged as the longest and among the warmest [20–22], as well as unusual, interglacial recorded in the last 800 ka [23].

## Methods

**Fig 1:**
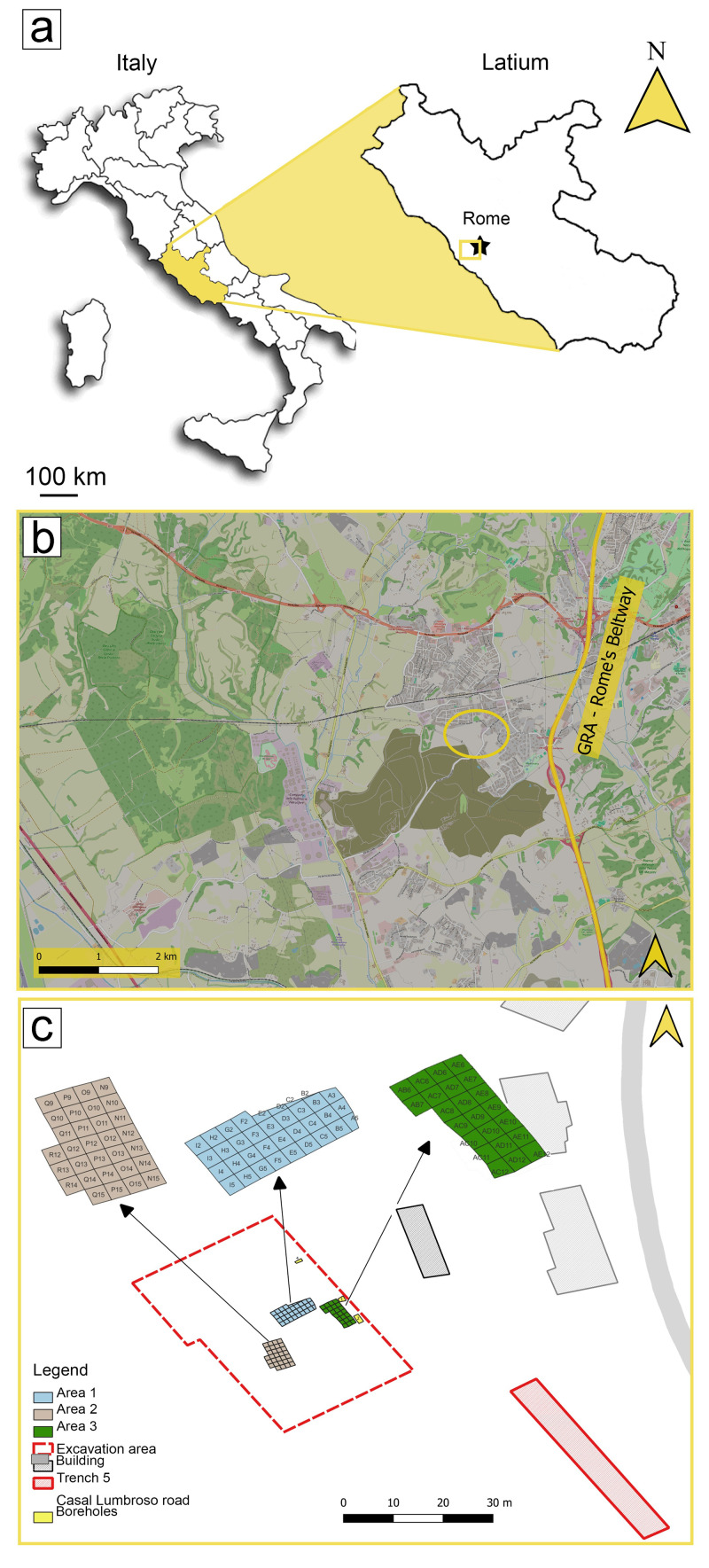
Geographic position of the archaeological and paleontological deposit of Casal Lumbroso (a,b) and position of the areas investigated during the fieldwork activities (c). Italian map (a) modified from https://it.wikipedia.org/wiki/File:Italy_map_with_regions.svg; map (c) taken from OpenStreetMap (https://osmfoundation.org).

In 2017, a rich archaeological and palaeontological deposit was discovered during the construction of a new building complex along via Casal Lumbroso, located in the north-west sector of Rome (central Italy) (Fig 1). The first excavations took place between 2017 and 2019, under the scientific direction of the Soprintendenza Speciale Archeologia, Belle Arti e Paesaggio di Roma. Fieldwork activities resumed in 2023, coordinated by Sapienza University of Rome, in collaboration with the Museo delle Civiltà (Rome) and the CNR-Istituto di Geologia Ambientale e Geoingegneria, and authorized by the Soprintendenza Speciale Archeologia, Belle Arti e Paesaggio di Roma.

**Major and minor elements tephra analyses.** In order to determine the tephra layers volcanic source, and/or equivalent eruptive units, sedimentological samples have been collected from SU1, SU3, and SU5b of the Casal Lumbroso deposits. Samples have been geochemically characterised in terms of major (> 1 wt%) and minor (< 1 wt%, > 0.1 wt%,) elements composition, by analysis of glass shards and micro-pumice fragments with the Electron Probe Micro Analyzer (EPMA).

The analysis has been performed with the EPMA at the CNR-IGAG, with a CAMECA SX-50 equipped with five-wavelength dispersive spectrometers (WDS). Operating conditions were set at 15 kV accelerating voltage, 15 nA beam current, and 10 μm defocused beam diameter to limit Na mobilization and loss, and 20 s element counting time for all elements. Wollastonite (Si and Ca), corundum (Al), periclase (Mg), magnetite (Fe), rutile (Ti), orthoclase (K), jadeite (Na), phlogopite (F), potassium chloride (Cl), baryte (S), F-apatite (P) and metals (Mn) were used as calibration standards. The Kakanui augite from the United States Geological Survey, was measured prior to the analytical run to evaluate the accuracy of the electron-microprobe analysis.

Data reduction and processing was carried out using the ZAF (Z = atomic number; A = absorption; F = fluorescence) correction, whilst data normalization and visualization were performed in Microsoft Excel®. We adopted 93 wt% as a threshold for the measured total: analyses with measured totals lower than 93 wt% were discarded. All compositional data are shown as oxide weight percentages (wt%) in the Total Alkali vs Silica [TAS; 24] classification diagram and bi-plots diagrams, with total iron (FeOt) expressed as FeO, and normalized to 100% on a volatile-free basis (i.e., excluding Cl) for correlation purposes.

**Excavation protocol.** During the preventive archaeology excavation conducted between 2017 and 2019, fossils, lithic and bone tools were recovered. These materials were carefully mapped, and a digital model of the surface was realized. Later, the palaeontological and archaeological remains were methodically removed from the site.

Systematic excavations were carried out during 2023. The palaeontological and archaeological samples comprise every remain with a length ≥ 2.0 cm – or smaller if diagnostic – recovered within a reference grid of one-square meter (1 m x 1 m) units. Each paleontological and archaeological remain is integrated into a Geographic Information System (GIS) architecture, which incorporates total station data, and a comprehensive database. Every specimen is labelled with a code (indicated with the initials RR followed by a progressive number) and linked to a database containing relevant information, including sector, square, stratigraphic unit, discovery date, material, preliminary determination, coordinates (x, y, z), axis direction, tilt, and other notes.

Three different areas were investigated (Area 1, 2, and 3; Fig 1), but archaeological and paleontological materials were mainly recovered from areas 2 and 3, encompassing approximately 28 square meters each. The fossils were all found within the same archaeological level.

**Palaeontology and bone taphonomy.** The osteological analyses of the large mammal fauna from the Middle Pleistocene site of Casal Lumbroso were carried out considering as a single assemblage remains collected from the 2017−19 excavations temporarily preserved at the Servizio di Bioarcheologia, Museo delle Civiltà, Roma (MUCIV), and the 2023 excavations temporarily stored at Laboratorio PaleoFactory, Dipartimento di Scienze della Terra, Sapienza Università di Roma (PF).

Taxonomic and skeletal element identifications are based on the reference collection of the MUCIV and PF. Because of fragmentation, in some cases it was not possible to assess species or higher taxonomic group, the specimens were then referred to one or more size groups. Considering the taxa identified at the site and those potentially living in the area during the relevant period, microfauna consists of microvertebrates such as small rodents, insectivores or herpetofauna; small ungulate may include roe deer and other ungulates of similar dimensions; fallow deer and red deer can be considered as medium ungulate; bovines and horses are in the large ungulate group; rhino and hippo are very large ungulates.

Measurements of the cranial, dental, and postcranial fossils were taken following von den Driesch [25] and Lister & Sher [26], with a digital caliper to the nearest 0.1 mm. Modified elephant bones were also measured.

All bone elements have been thoroughly inspected searching for human, carnivore, and other natural modifications; identifications of such modifications were done on the basis of current taphonomic literature ([27] and references therein) as well as of the MUCIV reference collection. Microscopic examinations have been carried out using a stereomicroscope (Nikon SMZ 1000), and a SEM (Tescan VEGA 3) at MUCIV.

**Lithic analysis.** The archaeological sample of Casal Lumbroso analysed here, collected from the 2017−19 excavations and the 2023 excavations are temporarily stored at the MUCIV and PF, respectively.

The archaeological record analysed consists of 542 lithic artifacts, supplemented by 7 found on the surface from areas exposed during the initial phases of excavation. The analysed lithic assemblage consists of 347 flakes, 54 tools, 16 cores, 1 hand-axe and 131 chunks/fragments. All the material originates from the same stratigraphic unit of the fauna.

We applied the *chaîne opératoire* method for the analysis of the lithic assemblage. According to this approach, the manufacturing of stone artifacts is viewed as a sequence of actions within a techno-economic system. The process that leads to an artifact is divided into technological stages: procurement, production, exploitation, use, abandonment and possible reuse.

The lithic assemblage is divided into techno-typological categories (i.e., flakes, cores, hand-axes, chunks/fragments, tools), for which the type of raw material, the percentage of cortex, the degree and the colour of patinas have been recorded. We meticulously documented both the occurrence and precise location of edge damage, alongside a comprehensive array of core and flake variables. Such variables include, but are not limited to, the number of striking platforms, the directions of flake scars, scar frequency, as well as the general classification of regular and irregular cores, cross-sectional profiles of flakes, termination patterns, platform morphology, bulb shape, and the count of both flake scar directions and dorsal scars.

**Residues and Use-Wear analyses.** A survey at the meso- and microscale of the unwashed chipped stone tools was firstly carried out with a digital microscope equipped with a reflecting light system (Hirox RH-2000, objectives with magnification range 35X to 2500X).

Residues were also extracted from various areas of the lithic items using a pipette and de-mineralized water as the medium. The extractions were then prepared on slide glasses and observed at both the mesoscale and microscale using the digital microscope Hirox RH-2000 equipped with a transmitted light system (for the residues protocol see also Lemorini et al. [3] and references therein).

Subsequently, the lithic items were initially washed with plain water and then successfully cleaned with de-mineralized water in an ultrasonic machine for 5 minutes. After drying, the lithic samples were observed at the mesoscale with a stereomicroscope (Nikon SMZ, oculars 10X, objective 1X, magnification range 0.75X–7.5X) and with the digital microscope Hirox RH-2000 (objective magnification range 35X–250X) to examine the edge removals. Finally, they were observed at the microscale using a metallographic microscope (Nikon Eclipse 600, oculars 10X and objectives 100X, 200X) equipped with reflected light to inspect the state of preservation of the micro-surface and detect any use-related polishes (for the use-wear protocol see also Lemorini et al. [3] and references therein).

**Isotope analysis.** The enamel samples from the elephant molar were collected at the MUCIV. The chemical pretreatment and isotope analysis were performed at CNR-IGAG Stable Isotope Laboratory).

The enamel samples were obtained from a fragmented left lower third molar of an adult elephant (inventory number 59). The sampling strategy was aimed to generate an intra-tooth isotopic profile; therefore, a sequential sampling of the fifth lamella, counting from the distal portion of the tooth, was performed, obtaining 16 samples along the tooth growth axis in 2–3 mm intervals. The tooth cementum over the area of interest was mechanically removed by drilling with a Dremel borer to expose the enamel. In addition, the uppermost surface of the tooth enamel was drilled away, to remove impurities and minimize the risk of sample contamination.

The chemical pretreatment was performed to remove organic matter and exogenous carbonate, following the procedure described by Bocherens et al. [28] and Koch et al. [29]. The enamel powder was soaked with 2.5% NaOCl for 24 h at 20°C, rinsed three times with distilled water, and then treated with 0.1 M buffer acetic acid-calcium acetate for 24 h at 20°C. The samples were rinsed repeatedly with distilled water and dried for 48 h at 40°C to remove any remaining water.

The carbon and oxygen isotope compositions of the samples were determined on the carbonate component of the tooth enamel using isotope ratio mass spectrometry. About 2 mg of treated powder was reacted with 100% phosphoric acid at 72°C with a Thermo Scientific™ Gas Bench II device connected to a Thermo Delta Plus mass spectrometer. The results were reported in the usual delta notation (δ^13^C and δ^18^O), representing the relative deviation in parts per mil (‰) with respect to the VPDB (Vienna Pee-Dee Belemnite) and VSMOW (Vienna Standard Mean Ocean Water) international standards, for carbon and oxygen respectively. Standardization was conducted using three laboratory standards calibrated against international standards NBS-18 and NBS-19. The standard internal precision of isotopic analysis for δ^13^C and δ^18^O was ± 0.1‰.

## Results

### Geological and chronostratigraphic setting

The archaeological and palaeontological deposit of Casal Lumbroso was accidentally discovered during the construction of a new building complex, when an elephant tusk was partially exposed by an excavator in 2017. The preventive excavations, requested and under the scientific direction of the Soprintendenza Speciale Archeologia, Belle Arti e Paesaggio di Roma, were conducted between 2017 and 2019. During these preliminary activities, animal fossil remains, lithic and bone tools were mapped, and a digital model of the depositional surface was realized. The palaeontological and archaeological remains were then methodically removed from the site.

Fieldwork activities resumed in 2023, coordinated by Sapienza University of Rome, in collaboration with the Museo delle Civiltà (Rome) and the Istituto di Geologia Ambientale e Geoingegneria, Consiglio Nazionale delle Ricerche, and authorized by Soprintendenza Speciale Archeologia, Belle Arti e Paesaggio di Roma. During these systematic excavations further remains were collected. Only the sediments of the 2023 excavations were sieved allowing the collection of a larger assemblage of unidentified fragments compared to the 2017–2019 activities.

Three different areas were investigated, but the archaeological and paleontological material was found only in one stratigraphic level, the same documented in 2017–2019 excavations.

**Fig 2:**
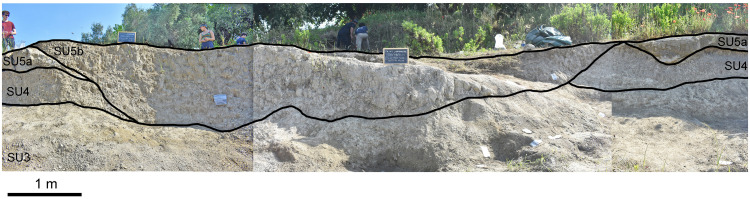
Stratigraphic succession of the Casal Lumbroso site. SU – Stratigraphic unit.

The sedimentary succession recognized in the area consists of fine, silty-fine sandy sediments with intercalated primary and sub-primary pyroclastic layer and faintly pedogenized horizons. Specifically, five stratigraphic units were identified, with the uppermost SU5 unit distinguished in two sub-units, from the bottom to top (Fig 2):

SU1: It is a sub-primary fallout deposit embedded in the silty-sandy sediment and made of white centimetre-sized pumices with abundant sanidine crystals.

SU2: It consists of greyish and massive silty-sandy sediment that develops upward into a pedogenized horizon.

SU3: It is a sub-primary fallout volcanic layer, few cm in thickness, laying on the pedogenized horizon (SU2) above SU1, made up of white centimetre-sized pumices and sanidine crystals.

SU4: It is a massive, white-greyish silty-sandy deposit topped by a pedogenized horizon.

SU5: This horizon is a massive grey volcanoclastic ash deposit, including rare centimetre-sized white pumices and abundant black-grey scoria, dark lithics, and free-crystals of leucite and pyroxene. It is disguised in two sub-units: a basal harder and more cohesive sub-layer (SU5a) and a top, more friable sub-layer (SU5b) separated from SU5a by an evident unconformity surface.

**Fig 3:**
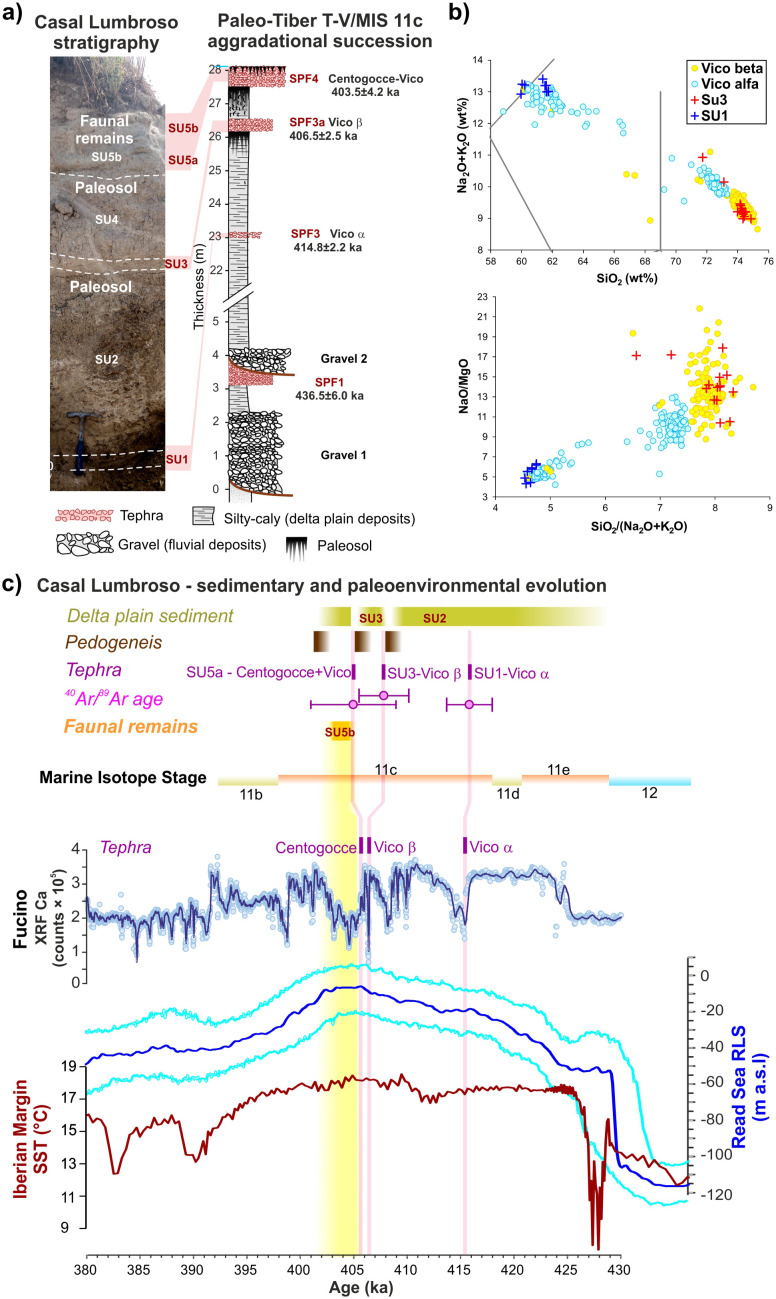
Stratigraphy, chronology and palaeoenvironmental setting of the Casal Lumbroso site. **a)** Sedimentary succession cropping out in the area surrounding the Casal Lumbroso site with the position of the two primary volcanic layers SU1 and SU3 and the volcanoclastic level SU5 which allow a correlation with the Paleo-Tiber aggradational succession of the glacial termination V (T-V) and marine isotope stage (MIS 11) [30]. **b)** Selected chemical bi-plots showing the glass composition of the Casal Lumbroso tephra SU1 and SU3 compared with Vico α and Vico β [31]. **c)** Chronological and paleoenvironmental framework of Casal Lumbroso sedimentary succession synchronized with the Fucino palaeolake Ca record, containing Vico α, and Vico β, and Centogocce tephra [32] and comparison with relative seal level (RSL) record based on the “Red Sea method” [33] and with the Iberian Margin Sea surface temperature (SST) variability at the Iberian Margin core MD03-2699 [34].

The faunal and archaeological material was found at the base of a reworked volcanic horizon, identified as stratigraphic unit (SU) 5b (Fig 3). By observing the stratigraphic sequence, it is evident that the deposit can be interpreted as a portion of palaeo-surface referable to the bed of a small water course, cut into a bank of compact volcaniclastic level (SU5a) and a clay level (SU4), and overlapping the second volcanic deposit (SU3). The remains were mainly found on the bottom of the bed. The abundance of the elephant remains referable to a single carcass found relatively close together and the virtual lack of traces of water transport on the specimens indicate the absence of transport for these remains. Other mammals were sporadically found, especially around the main concentration of elephant bones. Besides the elephant bones, other findings of relatively large dimensions are the cervid antlers, these too were found in a fragmented state but without signs of long transport. These observations would suggest a rapid burial of the remains after their final deposition.

In summary, the archaeological and palaeontological samples of Casal Lumbroso were collected from SU5b, indicating the presence of a relatively short-term depositional event, mainly related to the exploitation of an elephant carcass by humans.

### Geochemical analyses

**Tephra layers major and minor elements composition.** SU1 – It has a fairly homogeneous composition, and in the TAS diagram, it can be classified as a trachyte-phonolite (Fig 3), with a mean SiO_2_ content of 61.0 ± 1.6 wt%, whilst the alkali sums (i.e., Na_2_O+K_2_O) range between 12.9 and 13.4 wt%. The alkali ratio (i.e., K_2_O/Na_2_O) is very high, always > 2 (i.e., ultrapotassic) and up to 3.6, with a mean CaO/FeO ratio of 0.93 ± 0.07 and a low Cl content of 0.06 ± 0.03 wt% (error expressed as 2s standard deviation).

SU3 – It is rhyolitic in composition (Fig 3), with a mean SiO_2_ content of 74.1 ± 1.5 wt%, alkali sums of 9.4 ± 1.0 wt%, and a high alkali ratio ranging between 1.9–2.7. The CaO/FeO ratio ranges between 0.78–1.04, with a mean Cl content of 0.22 ± 0.07 wt%.

**Tephra identification, stratigraphic correlation and chronological framework** SU1 and SU3 tephra layers are characterized by high K_2_O/Na_2_O ratios, which are typical for the potassic-ultrapotassic rocks of the Quaternary Italian volcanoes [35]. SU3 is rhyolitic in composition, with an ultrapotassic (K_2_O/Na_2_O > 2) composition. This feature is distinctive of the Vico volcano, central Italy, where deposits of the eruptions from the so-called Vico period-I [36] activity are characterized by K-rich rhyolitic rocks [37]. Comparison between SU1 and SU3 with products from Vico volcano shows a good geochemical match with Vico α and Vico β, respectively (Fig 3). These two eruptions are similar in composition but, as highlighted in Pereira et al. [31], can be distinguished using proper major element ratios such as the CaO/MgO and SiO_2_/(Na_2_O+K_2_O) (Fig 3). The Vico α and Vico β eruptions have been dated respectively at 414.8 ± 2.2 ka and 406.5 ± 2.5 ka [32] and can be employed as important tephra marker horizon due to their distinctive composition, precise ages, and regional distribution [32].

In the local framework of the Tiber River aggradational successions, formed as stacked morpho-stratigraphic units in response to the Middle Pleistocene glacio-eustatic sea level oscillations, and specifically during the glacial termination sea level rise and the interglacials sea level highstands [38], Vico α and Vico β pumice falls represent important stratigraphic and chronological markers for the so-called San Paolo Formation [SPF; 39] formed during the T-V and MIS 11c highstand [37]. Within this sedimentary succession, the tephra correlated to Vico α and Vico β eruptions are termed SPF-3 and SPF-3a, respectively (Fig 3). Based on its stratigraphic position, at the top of the Casal Lumbroso succession, and especially its lithological features, the volcanoclastic unit SU5a can be reliably identified as the SPF-4 unit, which in the SPF aggradational succession represents the topmost level (Fig 3). Indeed, SPF-4, like SU5a, is composed by greyish volcanoclastic material containing juvenile fragments with a bi-modal composition of mixed trachytic-white pumices and foiditic-dark scoria, material respectively coming from an undefined Vico eruption and from the so-called Centogocce Fall sequence of the Colli Albani volcano [37,38]. A ^40^Ar/^39^Ar dating of the SPF-4 layer yielded the age of 403.5 ± 4.5 ka [37]. As discussed in literature [30 and 31], though reworked, the age of the volcanic material SPF-4 can be considered as very close to its deposition and hence of the deposits bearing faunal remains, and in any case within the uncertainty of the ^40^Ar/^39^Ar dating, because of the following reasons:

(i) in SPF-4 there is no crystal younger than ca. 400 ka. Considering that at that time the explosive volcanic activity of the Latium volcano, forming the catchment of the low course of the Tiber River, was very intense and continue, the lack of crystals coming from eruptions younger than Cento Gocce means that the fossiliferous sediment deposited immediately after these events.
(ii) more importantly, the fossiliferous sediment conformably lays at the top of the San Paolo formation aggradational succession, formed in response to the sea-level rise and highstand during the glacial termination V and the MIS 11c interglacial. This means that the sediments were deposited during MIS 11c. Indeed, if the fossiliferous sediment were deposited significantly after the MIS 11c high-stand, i.e., after a relatively long time elapsed since Cento Gocce-Vico eruptions, then the fossiliferous sediment would not be deposited within the San Paolo formation, as the subsequent MIS 11a to MIS 10 sea-level low stand, started around 395 ka, would lead a deep incision of the MIS 11c deposits and the formation of a glacial delta below the present sea-level, no longer accessible for us. Consequently, the age of the fossiliferous deposits has to be in any case within the MIS 11c, i.e., not younger than 395 ka. Therefore, the dated crystals in SPF-4 provides a temporally strict *terminus post quem* for the deposition of the SU5a and the subsequent sub-unit SU5b, which thus can reliably be dated to an age slightly younger than 403.5 ± 4.5 ka.

In addition, identification and correlation of the Casal Lumbroso tephra layers allow direct synchronization with the Fucino Ca record in central Italy, containing Vico α, and Vico β, and Centogocce tephra, and thus framing the investigated succession within the regional palaeoclimatic setting provided by this record (Fig 3) [32].

### Palaeontological and archaeozoological analyses

**Fig 4:**
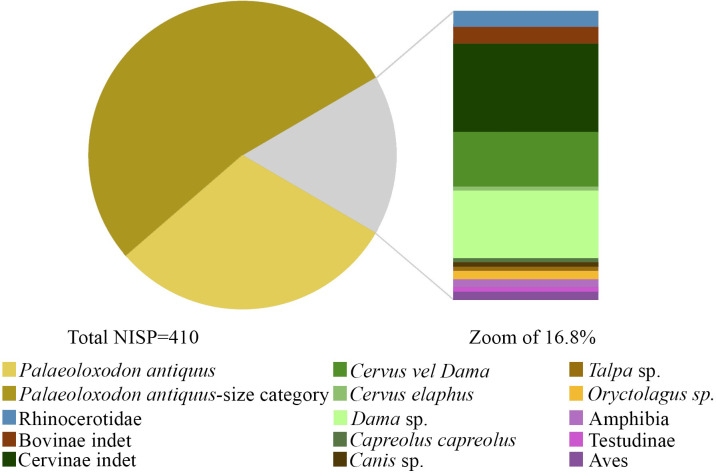
Vertebrate remains identified in the SU5a of Casal Lumbroso.

**Fig 5:**
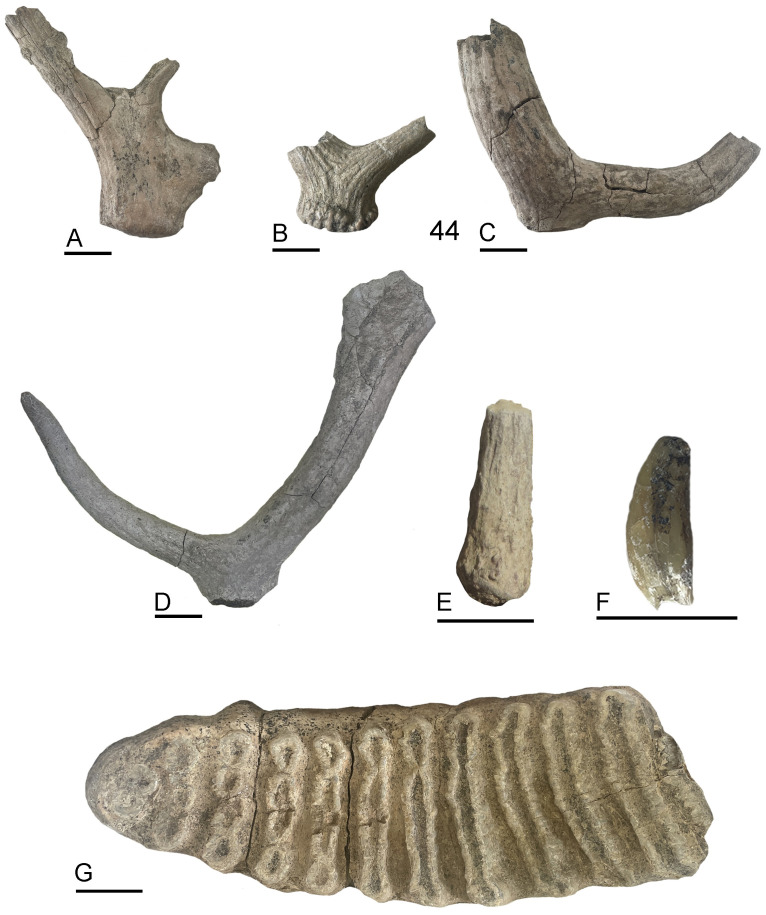
Fossil remains from Casal Lumbroso. A – 8, right antler of *Cervus elaphus*; B – 37, left antler of *Dama* sp.; C – 44, left antler of *Dama* sp.; D – RR164, right antler of *Dama* sp.; E – SN1, antler of *Capreolus capreolus;* F – SN16, left upper canine of *Canis* sp.; G – 59, left lower third molar of *Palaeoloxodon antiquus.* Scale bar 3 cm.

The faunal assemblage consists of 243 remains found during the 2017–2019 excavations and 2494 from the 2023 campaign (Figs 4–5 and S2; Table 1). Most of the sample is represented by unidentifiable remains (82.4%), 2.7% is referred to small to very large-sized ungulates, while the rest can be attributed to better defined taxonomic groups (Table 1). An isolated fossil was ascribed to Testudinidae, while the two bird specimens could be attributed to Anatidae and Strigiformes. Only two microvertebrate remains have been found during the sediment washing and picking, but they lack elements for a more precise identification (Table 1).

**Table 1:** Fossil remains from Casal Lumbroso. Abbreviations: NISP – Number of identified specimens.

	Repository			Total
**Museo delle Civiltà**	**Sapienza University of Rome**	
**Vertebrates**	**NISP**	**NISP**	**NISP**	**%**
*Palaeoloxodon antiquus*	151	190	341	12,46
Rhinocerotidae	2	2	4	0,15
Bovinae indet	1	3	4	0,15
Cervinae indet	10	11	21	0,77
*Cervus vel Dama*	12	1	13	0,47
*Cervus elaphus*	1		1	0,04
*Dama* sp.	13	3	16	0,58
*Capreolus capreolus*	1		1	0,04
*Canis* sp.	1		1	0,04
*Talpa* sp.		1	1	0,04
*Oryctolagus* sp.		2	2	0,07
Microfauna		2	2	0,07
Small/Medium Ungulate		3	3	0,11
Medium Ungulate	5	27	32	1,17
Medium/Large Ungulate		17	17	0,62
Large Ungulate	4	3	7	0,26
Large/Very Large Ungulate	2	13	15	0,55
Unidentifiable	40	2216	2256	82,43
Amphibia		2	2	0,07
Testudinae		1	1	0,04
Aves	2		2	0,07
**Total**	**243**	**2494**	**2737**	**100**

**Fig 6:**
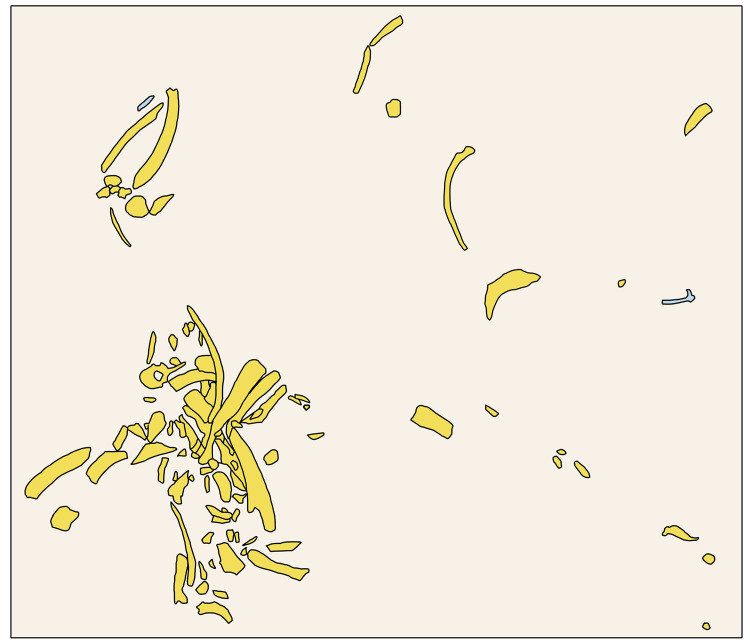
Distribution of large mammal remains of Casal Lumbroso. Color: orange – fossils of *Palaeoloxodon antiquus*; blue – fossils of *Dama* sp.

Among the mammal material identified in this work, most of the sample can be referred to  a straight-tusked elephant individual, *Palaeoloxodon antiquus,* about 45–49 years old (341 NISP; Figs 4-5 and S3; Table 1). These remains can be considered as part of a single carcass, and include a fragment of tusk, an isolated molar, a few nearly complete ribs, and a number of fragments of teeth, ribs, vertebrae, and especially diaphyses of long bones (*Palaeoloxodon*-size categories) (Fig 6 and S3; Table 1).

The elephant elements are mainly concentrated within a relatively small area (Fig 6) and belong to different parts of the skeleton (Fig S3), although some portions appear to be underrepresented. Tusks are incomplete but show a general straight outline. The lower third molar is hypsodont and possesses a high number of laminae, a high lamellar frequency, reduced enamel thickness and less developed cementum. These features are typically identified in *P. antiquus* [39,40,41].

Bovinae at Casal Lumbroso are documented by 4 specimens of scarce taxonomic value (Table 1). The specific identification of the Middle Pleistocene remains of bovids is a difficult matter, especially when only a few isolated specimens are available. During this period, three different forms were widespread in Europe, *Bos primigenius*, *Bison priscus* and *Bubalus murrensis*. In addition to dental fragments, bovines are documented by a large cuneiform, whose taxonomical identification at specific level is quite impossible [42 for discussion]. The sample is therefore referred to as Bovinae indet.

The presence of cervids is attested by 52 remains, including fragments of antlers, teeth, vertebrae, and long bones (Table 1). These fossils can be confidentially referred to as Cervinae indet.

Material clearly referable to *Dama* are fragmentary antlers, two dental fragments and a scaphoid. Five antlers can be referred to juvenile individuals, while the others are too fragmentary for the estimation of the age class. All the antlers with the preserved base are shed. The taxonomy of the Middle Pleistocene fallow deer is mainly based on antler morphology [43 and reference therein]. At Casal Lumbroso, the antlers preserve only the basal portion, with the burr, the beam, and, rarely, the first tine. The sample is therefore referred to as *Dama* sp.

Only an antler fragment can be referred to *Cervus elaphus* (Table 1). It consists of a burr and a few centimeters of a beam; this antler is shed. The size of this specimen (antero-posterior breadth of the burr 49.0 mm and antero-posterior breadth just above the burr 52.5 mm) falls in the variation of red deer. As observed for *Dama* antlers, also this fossil can be referred to as a juvenile individual.

A basal fragment of a shed antler documents the presence of *Capreolus capreolus*, belonging to a juvenile individual.

Rhinoceroses are documented only by 4 teeth fragments (Table 1). They are broken and poorly preserved, but the pattern of the enamel shows the typical arrangement observed in the rhinoceroses.

The only carnivore species documented at Casal Lumbroso is the wolf. The fossil is a portion of upper canine that can be referred to the genus *Canis*. During the mid Middle Pleistocene, the *Canis mosbachensis*–*Canis lupus* transition occurred [44]. These forms share many morphological features and show a discrete size overlap [45]. Considering this, the finding of Casal Lumbroso is ascribed to *Canis* sp.

Only a lower molar and a calcaneum can be referred to Lagomorpha, whose morphology and size can be referred to *Oryctolagus* sp. [46].

The presence of *Talpa* sp. is documented by an isolated fragmentary humerus.

No cut marks have been detected on the faunal assemblage, but some of the surfaces have been partially altered by sediment abrasion and limited water transport and this may have hampered the observations. Several animal remains, especially *Palaeoloxodon* specimens, show fresh bone fractures and in some cases also impact marks (see Bone artifacts section). Very few specimens (an elephant long bone shaft fragment and the pelvis) display carnivore damage, suggesting that the bones had been rapidly covered by sediments.

The taphonomic evidence indicate that the *P. antiquus* carcass was probably exploited not only as a food source (e.g., meat, marrow), but also for acquiring raw materials as documented by the presence of intentionally fractured elephant bones with localized use wear traces, some of them also with flake removals.

### Lithic assemblage

**Fig 7:**
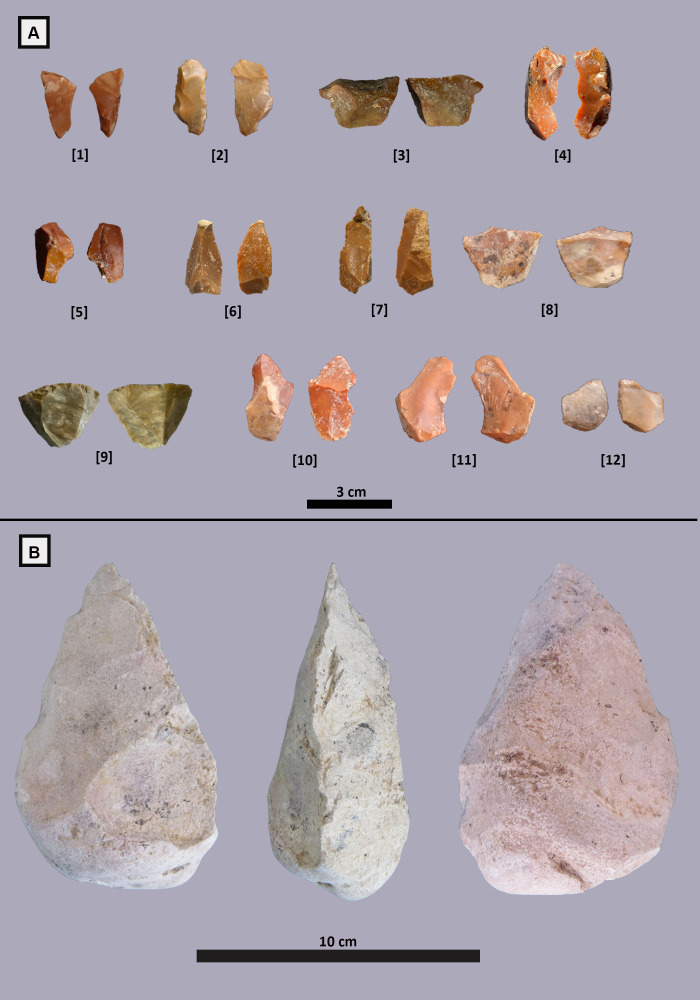
Lithic assemblage from Casal Lumbroso. **A)** Compression flakes (1,5,9); Flakes fragments; 2,4); Bipolar on anvil *débitage* flake; 6,7,11) double ventral flakes; 2,8,9) Flake with hinged distal part; 12) Flat flake. **B)** Limestone hand-axe.

The lithic implements were discovered in the same area with the concentration of the elements belonging to the elephant carcass. The analysed assemblage consists of 15 cores, 351 flakes, 54 tools, 1 hand axe and 131 chunks/fragments (Figs 7 and S4-5).

The lithic edges are preserved, showing little to no damage. Additionally, a patina is visible on approximately 70% of the lithic material (4% heavily patinated, 27% patinated, 37% slightly patinated). The most common colour of the patina is white (N = 310), followed by black (N = 79), which are respectively associated with post-depositional carbonation processes, and with manganese oxides and hydroxides. On some artifacts (N = 59), a double patina is observed, which could be related to pedogenesis processes and the potential reuse of the blanks over time.

The predominant raw material identified is flint, which may be found in the form of small pebbles near the site. These pebbles feature rounded edges and smooth surfaces, with an average weight ranging from 60 to 360 grams and typical dimensions of approximately 60 x 45 x 35 mm. Additionally, only ten flakes, one core and the single handaxe are made of limestone. These limestone artifacts tend to be larger on average compared to those made of flint, highlighting a potential size distinction between the two materials, well documented also at other Palaeolithic sites (e.g., Castel di Guido, Torre del Pagliaccetto [=Torre in Pietra) [3,12].

The bipolar-on-anvil technique [47–49] dominates the assemblage and is identified through flake features such as double ventral surfaces, some with distinct double Hertzian cones (Fig 7A, items 6, 7, 11), typically located on opposite surfaces yet sharing the same platform. Among the *débitage* products, bulb morphology is predominantly flat or normal (Fig 7A, item 12), with rare occurrences of ‘crown’-shaped bulbs and negative bulbs (Fig 7A, item 4). Flake terminations are frequently stepped (Fig 7A, items 1, 5, 6, 7) or hinged (Fig 7A, items 2, 8). Some flake fragments may derive from pre-existing pebble fractures or result from bending/compression forces between the hammer and anvil [49] or a soft anvil [50] (Fig 7A, items 1, 5, 9, 10). Additionally, it is possible to hypothesise that free-hand flaking was also employed, as suggested by the presence of pronounced percussion bulbs and convergent/centripetal flakes (Fig 7A 2, 3, 4). On the other hand, intermediate methods (i.e., combination of anvil and free-hand flaking) appear likely primarily based on the convergent and centripetal patterns observed on both simple cores and core-on-flakes (i.e., COFs). Furthermore, the use of *façonnage* is supported by the discovery of a highly eroded limestone hand axe (Fig 7B).

*Débitage* blanks reveal that five out of 15 cores were produced using COFs blanks, while other types are categorized as simple/opportunistic, angle change, and multidirectional (Figs S4-5). The axial recurrent and non-axial recurrent bipolar on anvil *débitage* flakes [50], which detach a volumetric portion of the pebble and have a sharp edge, were the most suitable products.

There are 142 unidirectional flakes, 7 bidirectional flakes, 104 convergent and 74 centripetal flakes.

The artifacts displaying traces of retouch are categorized as tools and their number is rather high (N = 54). At least some of the artifacts were retouched *in situ*, given the presence of 29 chips/retouch flakes. Retouch types are, in some cases, invasive and steep on the dorsal face (respectively 9% and 17%), while in most cases, dorsal and ventral shallow retouch scars are present.

The handaxe was discovered approximately 15 meters from an elephant carcass, suggesting a potential association with butchery or other subsistence activities. In terms of morphology, the handaxe measures 11.03 cm in length, 7.35 cm in width, and 4.86 cm in thickness, indicating a robust and compact design and as well the Elongation Index (EI = 1.50) and Refinement Index (RI = 0.66) the handaxe can be classified as short and thick ovate [51], or cordiform according to the typological framework established by François Bordes [52]. The butt of the handaxe is identified as the prehensile portion, suggesting it was the primary area gripped during use. The cutting edge of the handaxe is rectilinear and interrupted at the base. The tip itself is not particularly developed, indicating that the tool may have been designed for robust, forceful activities rather than precision work.

Among the formal tools, 21 denticulates (41%), 19 notches (35%), 13 scrapers, and one flake with irregular retouch were identified (Fig 7).

### Residue analysis

A macroscopic assessment of the whole lithic assemblage discovered during the excavation of the Casal Lumbroso site was conducted. This evaluation led to the selection of 22 items that were not visibly impacted by post-depositional processes (such as fractures, edge-damages, or weathering) and displayed sharp edges suitable for use.

**Fig 8:**
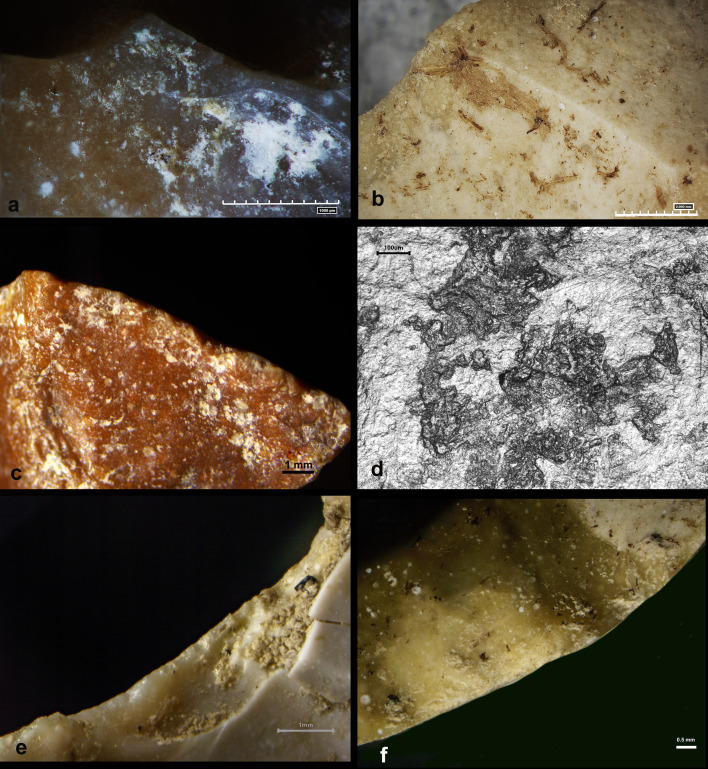
Lithic assemblage from Casal Lumbroso. a) Lithic tool showing whitish concretion on its surface; b) lithic tool showing residues of plant fibers due to contamination; c) detail at the mesoscale of edge-damages and edge-rounding due to weathering, possibly the slight rolling in a river flow; d) detail at the microscale of the alteration observed on the surface of the Casal Lumbroso lithic tools (widespread soil sheen marked by narrow and shallow striations due to abrasion processes); e) edge-removals by use, slightly overlapping, with a cone initiation and feather/step terminations and transversal direction showing an activity of scraping of soft-medium material (the edge removals are associate to an edge-rounding suggesting the working of a slightly abrasive material); f) edge-removals by use, with a bending initiation, a majority of feather terminations and slightly oblique direction showing an activity of cutting of soft material.

To investigate the presence of residues on the surface of the selected chipped stone tools, a survey at the meso- and microscale was first carried out. Contaminants of inorganic and organic nature such whitish concretions, remains of mold, roots and other vegetal fibres were documented (Fig 8a-b). The organic remains testify to a quite recent contact with humic soil.

An extraction was also performed using a pipette and de-mineralised water as the medium. The samples were prepared on glass slides and examined under transmitted light at the microscale. The residue analysis identified bimodal starch granules from the Triticeae tribe (Poaceae family) (rounded/oval, lenticular shape in 3D and central cross), which showed no signs of degradation, indicating that these residues are modern contaminants. No other residues were found, suggesting that post-depositional processes hindered the preservation of ancient residues.

### Use-Wear analysis

The mesoscale observation of the 22 lithic items revealed the widespread presence of small edge-damages combined with a slight edge-rounding possibly due to the weathering action of a slow water flow (Fig 8c). This type of mechanical alteration reduced the detection of edge-removals produced by use. However, in 13 cases, edge removals were clearly visible and not affected by the mechanical alteration. Traces related to use were documented on 5 flakes, 2 scrapers, 1 denticulate, and 5 slightly retouched flakes. Edge removals were observed on a single edge in all the lithic items, except for one.

The microscale examination of the lithic items revealed significant post-depositional alterations, likely due to abrasion by fine sediments. This led to a widespread soil sheen marked by narrow and shallow striations following the direction of the mechanical abrasion processes (Fig 8d). The intensity of these alterations hindered the observation of use-wear polishes, which were entirely removed.

Edge-removals revealed activities especially related to the scraping of materials of soft or soft-medium consistency with transversal motions (8 cases) (Fig 8e). In three other cases, cutting was detected on soft or soft-medium consistency materials (Fig 8f). The other two cases are related to a general use of soft-medium material.

### Bone artifacts

**Fig 9:**
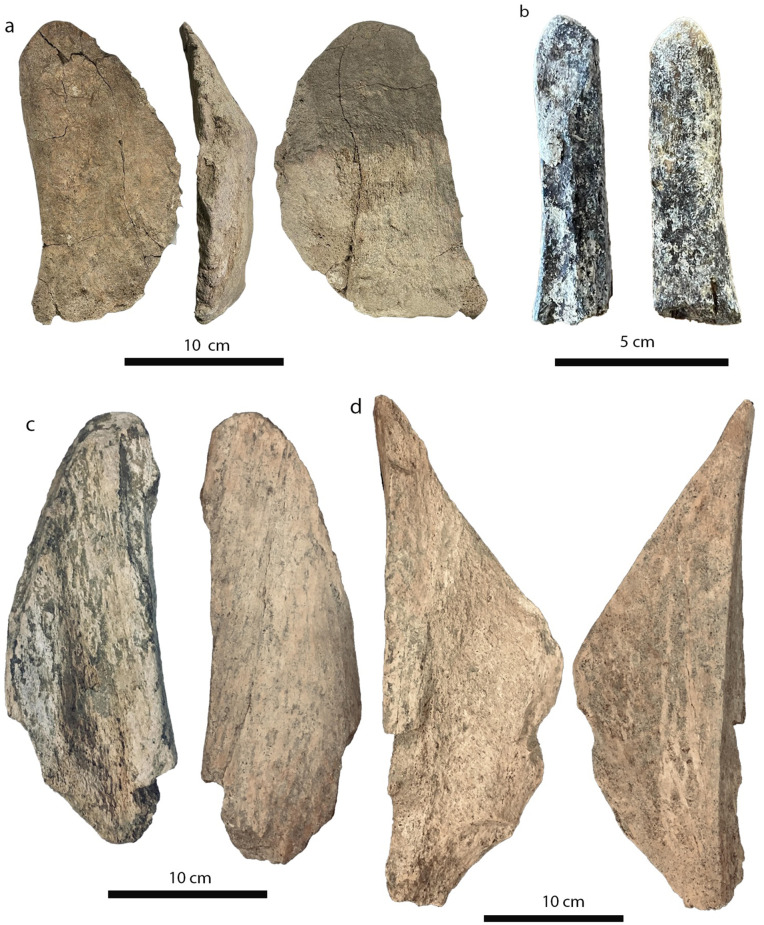
Selection of bone tools from Casal Lumbroso. a) large conchoidal elephant bone flake with localized use wear traces; b) large ungulate long bone diaphysis fragment with localized use wear traces; c) elephant long bone diaphysis fragment with localized use wear traces; d) elephant long bone diaphysis fragment with impact mark and localized use wear traces.

**Fig 10:**
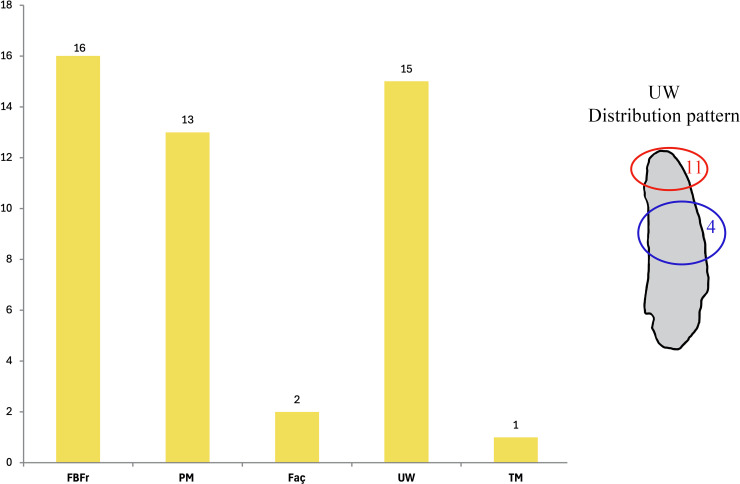
Distribution of the different specimens with categories of modifications. Abbreviations: FBFr – Fresh Bone Fracture; PM – Percussion Marks; Faç – Façonnage; UW – Use-wear, with localization on the fragments; TM – Tooth Mark. A specimen may present one or more modifications that were separately accounted for.

Sixteen elephant remains (ca. 15% of the *P. antiquus* assemblage), 13 long bone and three rib fragments (Figs 9–10), show evidence of fresh bone fracture and all of them, except one, also display localized use-wear traces; these latter are mainly concentrated on the tip of the specimens, while more rarely on the middle part of the fragment. Percussion marks produced for bone breakage were identified on 13 elements, while only two display evidence of *façonnage*, in one case with at least 10 removals along the edges.

Besides the modified elephant bones there is a single large mammal long bone with use-wear traces on the pointed tip (Fig 9b).

Although localized wear traces are macroscopically evident, preliminary microscopic investigations evidenced that the surfaces of the bones were often affected by natural processes and only in a few cases microscopic use-wear traces were preserved.

In general, the size of the used bone specimens ranges from approximately 10–36 cm with an average around 23 cm, therefore much larger than most of the lithic artifacts. Furthermore, there is a rib portion showing fresh bone fractures and use-wear traces on one end that reaches 77 cm.

### Isotope analysis

**Fig 11:**
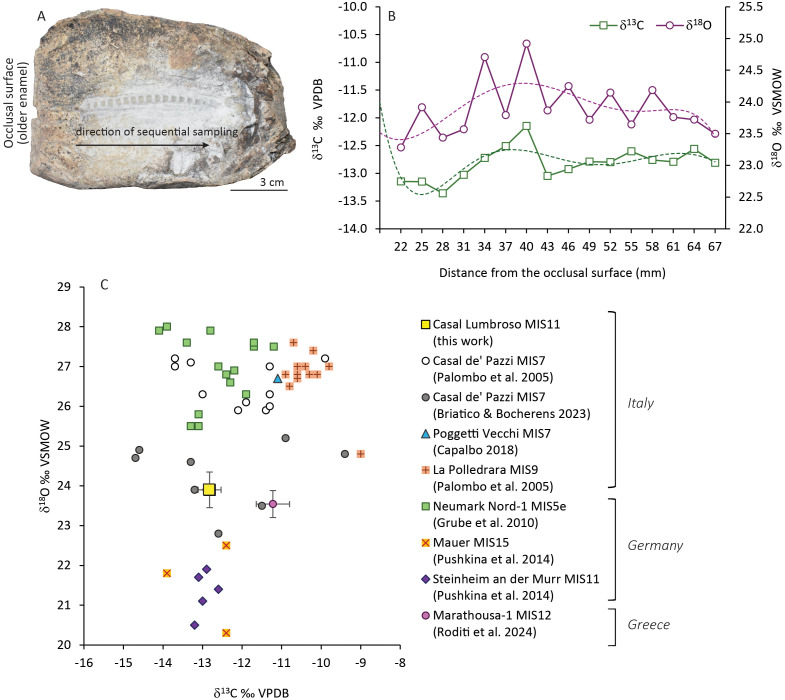
Isotope analyses on the elephant from Casal Lumbroso. a) photo of the *P. antiquus* tooth sampled for the isotopic analysis. b) intra-tooth carbon and oxygen stable isotope variability; dashed lines represent the polynomial regression curves. c) scatterplot of stable oxygen and carbon isotope compositions of tooth enamel from Casal Lumbroso and other Pleistocene European sites. For Casal Lumbroso and Marathousa-1 sites, the mean δ^13^C and δ^18^O values of the sequential samples are plotted (error bars represent the standard deviations).

The enamel of the elephant lower third molar was sampled for isotope analysis (Fig 11A). The δ^13^C values range between –12.15 and –13.36‰ VPDB (mean –12.82‰ VPDB) (Fig 11B; data in Table S2), indicating that the diet was composed entirely of C_3_ plants. The amplitude of isotope variation (1.21‰) indicates relatively low intra-tooth variability. However, a quasi-sinusoidal trend is observed in the intra-tooth profile and in the polynomial curve, with at least two downward and one upward period. The δ^18^O values range between +24.92 and +23.28‰ VSMOW (mean +23.90‰ VSMOW). As for the carbon, a low amplitude of variation (1.64‰) and a sinusoidal trend are observed also for the oxygen. The intra-tooth variations are supposed to reflect seasonal changes, although the low amplitude suggests that damping phenomena, due to enamel maturation and sampling procedure, could have occurred. Overall, the upward periods identified in the δ^18^O series, which coincide with peaks in δ^13^C values, likely reflect summer conditions, whereas measured minima in δ^18^O and the corresponding δ^13^C troughs express winter-time conditions. Considering that the molars of adult elephants form over several years and the number of recognized peaks in the intra-tooth profile, the isotope values possibly recorded season variations over a period of ~ 2 years.

The average isotope value of the tooth enamel of Casal Lumbroso has been compared with data of *P. antiquus* from several Pleistocene European sites, in order to evaluate the environmental and climatic variability among different sites and ages [53–58] (Fig 11C). With respect to the Italian sites, data suggest that the elephant of Casal Lumbroso foraged under greater closure and/or humid environments compared to Poggetti Vecchi, La Polledrara and several of the individuals from Casal de’ Pazzi. Elephants from Casal de’ Pazzi showed a wide range of isotope values, suggesting mixed-C_3_ feeding in both closed and open habitats [53]. The comparison with the other interglacial specimens from European sites is influenced by the different geographical context; in particular, the populations from Steinheim an der Murr and Mauer in Germany likely experienced cooler or more humid conditions due to higher latitude and continentality effects, whereas in Neumark Nord-1 the unexpected high oxygen isotope values are explained as a site effect (i.e., the presence of a lake, possibly affected by evaporation, as a source of drinking water) [55]. The elephant from Marathousa-1, a glacial site (MIS 12) located in Greece, experienced a more open C_3_ habitat with water-stress and/or slightly cooler conditions in comparison to the elephant from Casal Lumbroso.

Overall, data suggest the diffusion of humid and forested environments in Europe during the interglacials MIS 15 and MIS 11, whereas a degradation of the climate conditions and the evolution towards slightly more open landscapes may have occurred during MIS 7.

## Discussion

Casal Lumbroso documents a short-term occupation focused on the exploitation of an elephant carcass by humans about 400 ka ago, favoured by the mild climatic condition of the EMPT. The carcass, as suggested also for other Middle Pleistocene sites [12,59,60], may have belonged to an elephant that died of natural causes and was then exploited by hominins both as a food and a raw material source, and later partially dispersed and modified by natural agents (especially water), even though we cannot exclude the possibility that it was deliberately driven by humans to a natural mud trap [61]. The straight-tusked elephant is represented by fragments of tusk, an isolated molar, a few nearly complete ribs, a vertebra, some foot bones, and a partial pelvis as well as a number of fragments of teeth, ribs, vertebrae, and especially diaphyses of long bones, all of the size of an adult *Palaeoloxodon* (Fig S3; Table 1). The concentration of disarticulated elephant skeletal elements, likely from a single carcass, and the underrepresentation of some of them (e.g., skull, vertebrae) or portions of them (e.g., long bone epiphyses), may be attributed to different taphonomic agents. Considering the depositional context, water surely played a role, but humans may have been important as well; in this regards bone breakage for marrow extraction and raw material procurement is documented at the site. Furthermore, besides possible natural causes, the almost complete absence of the cranial portions could be referred to human exploitation of this nutritionally rich element that may have also been transported elsewhere [62]. The presence of a single elephant carcass with a dispersion of skeletal elements, but still within a limited area, has been documented also at other European sites such as Aridos 1 (Spain), Ficoncella (Italy), Southfleet Road (UK) [63–65]. Elephant butchery sites in the Early and Middle Pleistocene have been mainly discovered in fluvial-lacustrine depositional contexts, as for example Castel di Guido [12,60] or Poggetti Vecchi [66]. Haynes [67] noted that the accumulation of elephant bones in fluvio-lacustrine environments is not a mere coincidence since these animals often wandered into muddy swamps where they eventually became trapped, making the ideal environment for human food provisioning. Several examples are known, but, as far as Italy is concerned, the most iconic one is La Polledrara di Cecanibbio, where at least three individuals died trapped in muddy sediments [14,59 and references therein] and were then exploited by hominins. In the case of Casal Lumbroso, stratigraphic, palaeontological, zooarchaeological, taphonomic, and archaeological analyses suggest a depositional event occurring over a relatively short time frame in a fluvial context. Indeed, stratigraphic and taphonomic data indicate that archaeological and palaeontological remains were deposited on the bed of small water course, cut into a bank of compact volcaniclastic level (SU5a) and a clay level (SU4). The abundance of elephant remains refers to a single carcass found close together, and the lack of signs of water transport on the specimens indicates absence of transport for such remains, and that the animal died on the bed of the stream, close to the bank. Correspondingly, the associated lithic industry does not present signs of fluvial transport (e.g., no signs of water transport, no smoothed edges, etc.). Most of the faunal remains are highly fragmented and undeterminable at the taxonomic level, but again with very limited traces of water transport. Palaeontological analyses revealed the presence of several species that were a potential food source for the pre-Neanderthal population such as Rhinocerotidae, Bovinae indet., Cervinae indet., *Cervus elaphus*, *Dama* sp., *Capreolus capreolus*, in addition to *Palaeoloxodon antiquus*. An evidence that would suggest a relatively short depositional event is the seasonality as indicated in particular by Cervid remains: fallow deer, red deer and roe deer are primarily represented by antler remains and all the portions with preserved bases belong to shed elements: five shed antlers for fallow deer, one for red deer, and one for roe deer. In modern red deer, antlers are shed between late January and April, in fallow deer, this occurs between late March and early June, while in roe deer it happens between October and November. Considering the prevalence of fallow deer and red deer shed antlers, the deposition of the Casal Lumbroso faunal assemblage probably occurred mainly at the end of the spring. The isotope analysis carried out on the enamel of the lower third molar of *P. antiquus* and the comparison of these data with other European contexts, revealed the presence of wooded environments and humid climatic conditions at Casal Lumbroso. The intra-tooth variations of isotope compositions documented seasonal changes, although with low amplitude oscillations in both δ^18^O and δ^13^C, in which the lower measured values correspond to winter-time conditions and *viceversa*. Albeit limited in scope, due to the scarce taxonomic value of the identified remains, the presence of cervids (*Cervus elaphus*, *Dama* sp., *Capreolus capreolus*) appears to confirm the presence of wooded environments around Casal Lumbroso.

Archaeological analyses allow to ascribe the lithic artifacts, mainly made of flint, to the late Acheulean, an age compatible with the chronological framing of the site. The sediments where the archaeological and palaeontological remains were collected did not contain lithic raw materials, however the small pebbles were probably found in outcrops near the site, like in the Ponte Galeria Formation, where several authors documented the presence of pebble outcrops [68,69].

The lithic implements of Casal Lumbroso, as previously mentioned, are small in size, nevertheless the assemblage includes denticulates, notches, scrapers, and one handaxe as well as many usable flakes, most of which were probably knapped *in situ* possibly for the exploitation of the elephant carcass. Percussion on anvil was largely used to overcome the small dimensions of the pebbles, and cortical flakes and *calottes* are common, quite similar to the assemblage recovered at Capanna Murata [70,71]. Recently, small lithics items (< 30 mm), generally defined as “small tools” or “small flakes”, have attracted renewed interest among archaeologists. Small tools can be separated into two categories based on size, the first between 5 and 15 mm, and the second between 15 and 30 mm [72]. Experimental data demonstrate that small flakes play a significant role in processing soft animal tissue, especially in handling the fat portion of the carcasses [73]. The abundant use of small flakes in the butchery site appears related to their highly precise cutting actions [72]. Small flakes have also been found in association with elephant carcasses at several sites, where use-wear analysis confirmed their role in butchery activities [72,73]. Combining their small size, efficiency in cutting activities and morphological variability (which reflects their use in a variety of specific tasks), small flakes have become relevant components of the ideal toolkit for mobile communities [33 and references therein]. The lithic assemblage from Casal Lumbroso is predominantly composed of small flakes ranging in size from 5 to 30 mm, which constitute 62% of the sample and 86% of the flakes (Table S3). The use-wear analyses indicate their use on soft or soft-medium consistency materials, compatible with butchery. Overall, the lithic industry from Casal Lumbroso perfectly fits into coeval technological methods and processes studied in Latium. However, the presence of a limestone handaxe, suggests that larger tools could be effective in order to process the carcasses (e.g., for the bone breakage that is indeed observed in the faunal record) [74,75]. The handaxe could have also played a role in the manufacture of the bone tools identified on site. The presence at the same time of small tools, limestone and bone tools is indeed one of the signatures of Italian Middle Pleistocene sites [75].

**Fig 12:**
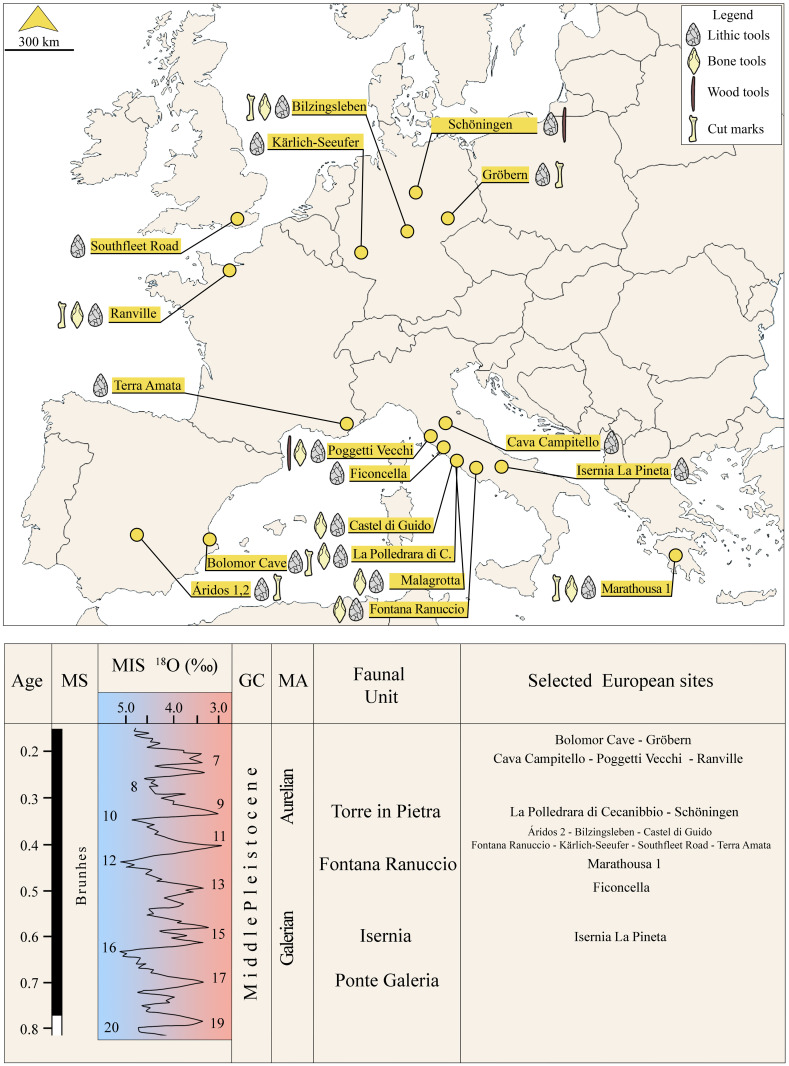
Geographic distribution of the elephant butchery sites from Europe and their chronological context. Map of Europe modified from https://en.wikipedia.org/wiki/File:Europe_blank_map.png.

Methodological approaches introduced at the beginning of the 2000s discussed the necessity to consider several elements to validate the interpretation of a fossil accumulation containing lithic artifacts as a butchery site [2,8]. Indeed, the mere spatial association between lithic tools and an elephant carcass is no longer considered valid evidence of a pre-depositional interaction between hominins and these very large animals [2,8]. Based on the new criteria, the interpretation of some of the previously iconic elephant butchery sites such as Torralba and Ambrona in Spain [76–78] and Notarchirico in Italy [79] has been invalidated.*Palaeoloxodon* from Casal Lumbroso does not exhibit cut marks, but this is not unusual, since cut marks have rarely been identified in European butchery sites, and their absence does not necessarily exclude human involvement (e.g., Áridos 2, Spain [19], Bilzingsleben, Germany [80], Bolomor Cave, Spain [81], Gröbern, Germany [82], Marathousa 1, Greece [83], Ranville, France [84]; Fig 12). Their presence or absence may depend, for example, on cutting depth, speed of the butchery process, and types and size of the tools used, as well as on the large amount of meat in megaherbivores that would make it difficult to reach the bone with the tool [8–10]. Cut marks are therefore in general rare on elephant carcasses at Pleistocene butchering sites, besides other factors their absence could also be related to the size of the tools used. Since small tools are a significant component of the lithic assemblages in these contexts, sometimes representing over 80% of the sample, it must be taken into account that smaller tools produce lower frequencies of cut marks on the bones compared to larger artifacts, as indicated also by experimental analyses [85].

When cut marks are absent, other evidence may help in the identification of a butchery site, such as, for example, use-wear and residue analyses on the lithic tools associated with the carcass, which could document the actual use of such tools in animal processing [2,3,86]. At Casal Lumbroso, the absence of residues and preserved polishes, which would have allowed for a more detailed identification of the worked material, prevented a direct link between the analysed lithic tools and butchery activities, providing only more general, circumstantial information, suggesting the tools were primarily used for treating soft or soft-medium materials.

Other elements therefore should be taken into consideration when elephant carcasses are found associated with lithic tools before determining whether these contexts represent butchery sites [2]. These include the stratigraphic and spatial distribution of the bones, the presence/absence of skeletal elements in anatomical connection/physiological position, and the analysis of the degree of disarticulation and their origin. The elephant carcass of Casal Lumbroso consisted mainly of ribs, vertebrae, pelvis, tusk and tooth, while the long bones are highly fractured with virtually no epiphyses present (Fig S3). Although these bones were not found in anatomical connection, they were all relatively concentrated, close to one another, within a small area of about 5 square meters.

In order to determine whether the bone fragmentation in Casal Lumbroso is to be attributed to human activity for marrow extraction or other factors, such as trampling or deriving from large carnivore activity [87,88], careful zooarchaeological and taphonomic analyses have been carried out. The study of the elephant sample evidenced several green bone fractures, indicating that the bones had been broken when fresh; however, only the associated presence of impact marks in almost all these specimens (13 out of 16), represents the unequivocal evidence of human-elephant interaction at this site. Furthermore, the occurrence of localized use-wear traces on all the specimens with fresh bone break except one, indicates that these bones were also used as tools (Fig 9). At Castel di Guido, particularly relevant for the study of Casal Lumbroso due to both chronological and geographical proximity, the bones were thoroughly fractured, and the epiphyses systematically separated from diaphysis [12,89]. Unlike other European sites, the elephant bones of Castel di Guido are not entirely preserved, with only elephant tusks, ribs and vertebrae being nearly complete [12,89], mirroring the observations at Casal Lumbroso.

In addition to being fractured and used, at least two elephant bones were also knapped by hominins, to produce more formal tools, as it happens in other Middle Pleistocene sites of Europe, such as Bilzingsleben [80], Marathousa 1 [4,83], Poggetti Vecchi [66] and Ranville [84] (Fig 12). This behaviour is repeated throughout the Middle Pleistocene in the Latium region [90], as documented at Castel di Guido [12,89], Fontana Ranuccio [91], La Polledrara di Cecanibbio [92,93], and Malagrotta [70] (Fig 12). At Castel di Guido, bone tool dimensions exceed those of stone tools, possibly a consequence of the general scarcity of the lithic raw material in this territory, because the local pebbles are relatively small [12]. This could be the case also for Casal Lumbroso where the size of the bone tools largely exceeds that of nearly all the lithic artifacts (except for the handaxe), whose dimensions are mostly lower than 10 cm with only 16 items above that size (Fig 7 and S4-5).

It is largely accepted that humans produced large bone flakes to be used for different activities [88 and references therein]. Recently, the presence of bone tools has also been documented in three other sites of the Latium region, not associated with elephant butchery, as Colle Avarone, Isoletta, and Selvotta, whose ages range between 430 ka and 410 ka [94]. The use-wear analyses carried out on specimens from these three sites indicate that these tools have been involved in both butchering activities and ground excavation. Unfortunately, a preliminary attempt to analyse the use-wear traces on the bone tools from Casal Lumbroso revealed that they were almost completely obliterated by natural processes, but future investigations may provide further indications about their use and test the hypothesis that they, or at least some of them, could have been used also for ground excavation as in the study by Marinelli et al. [94].

As presented in Supplementary Information, the north-west district of Rome is characterised by a number of sites yielding elephant bones modified by humans, lithic industries, and in some cases, flaked bone tools. The authors documented the presence of handaxes and bone tools and showcased a clear exploitation of flint pebbles, with many flakes retaining their cortex, as well as a notable abundance of retouched flakes and a relatively modest size of the tools. At La Polledrara di Cecanibbio, an elephant carcass was exploited using small flint pebbles, no handaxes were recovered, and some elements displayed fresh bone fractures believed to be the result of human activity for the extraction of marrow; few of them had also been modified as tools [3,93]. In the long sedimentary sequence of Torre del Pagliaccetto, the exploitation of small flint pebbles in part with the bipolar-on-anvil method, characterised by multidirectional and uni-bidirectional irregular cores was reported from the level ‘m’ [95]. Retouched flakes documented at Torre del Pagliaccetto are similar in shape and dimensions to those described here from Casal Lumbroso. At Ficoncella, a small flake assemblage was found, and the presence of retouched flakes provides direct evidence of *in situ* human activity [63]. Similarly, within the Latium region, sites such as Fontana Ranuccio [96], Isoletta [97], Campogrande di Ceprano [98], and Quarto delle Cinfonare [99], exhibit quite similar exploitation of small flint pebbles and similar technological lithic record, including the scanty presence of bifaces and the association with bone tools.

The stratigraphical, palaeontological, archaeozoological, and lithic data mentioned above all converge to place Casal Lumbroso within the broader framework of the Early-Middle Pleistocene Transition, where a clear pattern of pachyderm exploitation by pre-Neanderthals emerges, facilitated by mild climatic conditions and the presence of water streams, which ensured a predictable availability of fauna and possibly raw materials. Given the repeated occurrences of such sites during the MIS 13–7 interval of the Middle Pleistocene, particularly in central Italy, it appears that human groups followed a specific and long-lasting strategy aimed at the optimal use of available and predictable resources, including the complete exploitation of animal carcasses as sources of protein and fat, as well as osseous raw materials with long-lasting utility. The adaptation to the use of very small flint pebbles to produce small yet effective tools suited for butchering is part of this strategy. Despite some uncertainty regarding the human exploitation of other faunal remains at the site, the ecological and geographical setting of Casal Lumbroso during MIS 11 would have favoured human occupation by providing a certain degree of resource predictability. The Casal Lumbroso site thus contributes to expanding our understanding of human-animal interactions at a critical stage in human evolution and within a highly distinctive environmental context.

## Supporting information

S1 File Supplementary information. (DOCX)

S1 Fig Geographical location of the archaeological and palaeontological deposits of Rome. 1 – Aventino; 2 – Batteria Nomentana; 3 – Boccea; 4 – Campidoglio; 5 – Campo di Merlo; 6 – Casal de’ Pazzi, Ponte Mammolo; 7 – Castro Pretorio; 8 – Cava Nera Molinario; 9 – Monte Celio; 10 -Fondamenta BNL, Pincio, Quirinale; 11 – GRA 2 Km; 12 – Monte Antenne; 13 – Monte delle Pliche; 14 – Monte Mario; 15 – Monte Sacro; 16 – Monte Verde; 17 – Monti della Farnesina; 18 – Parioli; 19 – Ponte Molle; 20 – Porta Cavalleggeri; 21 – Porta Flaminia; 22 – Porta Pia, Porta Salaria; 23 – Prati Fiscali; 24 – Redicicoli; 25 – Sant’Agnese; 26 – San Paolo; 27 – Saccopastore; 28 – Sedia del Diavolo; 29 – Tor di Quinto, Cava Montanari; 30 – Via Aurelia; 31 – Via Cassia; 32 – Via del Tritone; 33 – Via Nazionale; 34 – Via Ostiense; 35 – Via Portuense; 36 – Vigna San Carlo; 37 – Vigne Torte; 38 – Villa Chigi; 39 – Capanna Murata, Pio Istituto di Santo Spirito; 40 – Castel di Guido; 41 – Cava Arnolfi, Cava Alibrandi, Muratella di Mezzo; 42 – Cava di Breccia di Casal Selce; 43 – Cava Rinaldi; 44 – Collina Barbattini, Via Aurelia 18.9 Km, 19.0 Km and 19.3 Km; 45 – Fontignano; 46 – La Maglianella; 47 – La Polledrara di Cecanibbio; 48 – Pantano di Grano; 49 – San Cosimato – Santa Cecilia; 50 – Via della Pisana; 51 – Torre del Pagliaccetto; 52 – Vitinia. Yellow star – Casal Lumbroso.
(PNG)

S2 Fig Archaeological and palaeontological deposit of Casal Lumbroso (a) and the draw of the large mammal bones exposed in the main surface (b). (JPG)

S3 Fig Skeletal elements of *Palaeoloxodon antiquus* from Casal Lumbroso. (JPG)

S4 Fig Lithic sample of Casal Lumbroso. Unidirectional recurrent (1,2), bidirectional recurrent (3), unidirectional on core on flake (4), centripetal on core on flake (5) and centripetal (6).
(JPG)

S5 Fig Lithic sample of Casal Lumbroso. Flint flakes: centripetal flakes (1,2), convergent flakes (3,8), bidirectional irregular (5,6), and unidirectional (7); Chert flakes: centripetal flake (4); Limestone flakes: cortical (9), unidirectional (10,11[refitting flakes]).
(JPG)

S1 Table Selected Middle Pleistocene mammal assemblages from Rome basin. (XLSX)

S2 Table Carbon and oxygen stable isotope values for 16 enamel sequential samples from the left lower third molar of the *Palaeoloxodon antiquus* individual from Casal Lumbroso. To evaluate the possibility of contamination from external carbonate sources, we assessed the carbonate content (CaCO_3_%) in the enamel. The range of CaCO_3_ in the enamel structural carbonates, from 4.7 to 5.8%, falls within the range of CaCO_3_ contents of modern enamel (~3.0 − 5.5%) (58) and ungulate enamel bioapatite (4.5 − 5.1%) (59).
(DOCX)

S3 Table Small *débitage* of Casal Lumbroso subdivision following Venditti et al. [41]. (XLSX)
