# Correction: From meat to raw material: the Middle Pleistocene elephant butchery site of Casal Lumbroso (Rome, central Italy)

**DOI:** 10.1371/journal.pone.0346584

**Published:** 2026-04-03

**Authors:** Beniamino Mecozzi, Ivana Fiore, Biagio Giaccio, Francesca Giustini, Stefano Mercurio, Lorenzo Monaco, Alessia Argento, Francesco Bucci Casari degli Atti di Sassoferrato, Isabella Caricola, Cristina Lemorini, Francesco Lucchini, Ilaria Mazzini, Maria Rita Palombo, Raffaele Sardella, Andrea Sposato, Enza Elena Spinapolice, Francesca Alhaique

The Funding statement for this article is incorrect. The correct Funding statement is as follows: This study was financially supported by the University of Rome, Sapienza (MUR – PRIN 2022FATAHZ - CUP B53D23001520006 to EES).
