# Supplementary material for: From meat to raw material: the Middle Pleistocene elephant butchery site of Casal Lumbroso (Rome, central Italy)
Source: PLoS One. 2025 Oct 8;20(10):e0328840. doi: 10.1371/journal.pone.0328840 (PMC12507280; doi:10.1371/journal.pone.0328840)
Supplement: S1 File — (DOCX) [file pone.0328840.s001.docx]

**Supplementary Information**

**From meat to raw material: the Middle Pleistocene elephant butchery site of Casal Lumbroso (Rome, central Italy)**

Beniamino Mecozzi^1,2*^, Ivana Fiore^1,3^, Biagio Giaccio^4^, Francesca Giustini^4^, Stefano Mercurio^5*^, Lorenzo Monaco^6^, Alessia Argento^7^, Francesco Bucci Casari degli Atti di Sassoferrato^5^, Isabella Caricola^8^, Cristina Lemorini^5^, Francesco Lucchini^5^, Ilaria Mazzini^4^, Maria Rita Palombo^4^, Raffaele Sardella^2^, Andrea Sposato^4^, Enza Elena Spinapolice^5^, & Francesca Alhaique^3^

^1^Dipartimento di Biologia Ambientale, Sapienza Università Di Roma, Rome, Italy

^2^Dipartimento di Scienze Della Terra, Sapienza Università di Roma, Rome, Italy

^3^Servizio di Bioarcheologia, Museo delle Civiltà, Rome, Italy.

^4^Consiglio Nazionale Delle Ricerche (CNR), Istituto di Geologia Ambientale e Geoingegneria, Area della Ricerca di Roma 1, Rome, Italy.

^5^Dipartimento di Scienze delle Antichità, Sapienza Università di Roma, Rome, Italy

^6^Consiglio Nazionale delle Ricerche (CNR), Istituto di Scienze Marine, Area della Ricerca di Bologna, Bologna, Italy

^7^Soprintendenza Speciale Archeologia Belle Arti e Paesaggio di Roma, Rome, Italy.

^8^School of Archaeology and Maritime Cultures, Haifa University, Haifa, Israel.

**The context of the “Campagna Romana”**

The urban area of Rome and its surroundings, known as “Campagna Romana”, is renowned as one of the most important territories for studying the Middle Pleistocene terrestrial ecosystems, owing to an exceptional abundance of palaeontological and archeological remains [1-4]. Many of these finds, dating to the end of the 19^th^ century and the early decades of the 20^th^ century, were discovered during major infrastructure projects that exposed extensive Pleistocene sedimentary sequences [3-4] Unfortunately, these sequences have been progressively destroyed or covered by anthropogenetic deposits. Other deposits were discovered during the 1950s and 1970s, particularly in the north-west sector of the city, near the Aurelia Statal Road and in the Ponte Galeria area. The extensive mammal record of the Roman Campaign plays a key role for biochronological correlations and reconstructing palaeoenvironmental conditions in the Italian Peninsula during the Middle Pleistocene [5-9].

The Middle Pleistocene of the Rome area was influenced by two main important factors: tectonics and recurrent glacio-eustatic fluctuations, coupled with the onset of the activity of the Roman Magmatic Province[3,4,10]. Volcanic products hold considerable importance, providing direct and indirect age determinations for numerous archaeological and palaeontological localities of Rome.

The significant contribution of the fossil record of Rome in the European context is evident in the naming of the Land Mammal Ages (LMAs), which were introduced by Italian palaeontologists, and later adopted throughout western Europe. The first of these, the Galerian LMA, derives its name from the Ponte Galeria area, where several deposits dating to the early Middle Pleistocene were discovered (e.g., Cava Arnolfi, Cava Alibrandi, Cava Breccia di Casal Selce, Cava Rinaldi). The second, the Aurelian LMA, was introduced by Gliozzi et al. [5] based on the rich record collected from various sites located along the Aurelia Statal Road, especially from Castel di Guido, La Polledrara di Cecanibbio and Torre del Pagliaccetto.

Nevertheless, it is worth noting that the majority of these fossil records was collected through archaeological and palaeontological surveys. Systematic excavations in the Rome area have been relatively scarce compared to the huge number of sporadic findings, both in historical time and last decades. The only site excavated using a systematic approach during the early 20^th^ century was Saccopastore, where fieldwork was conducted in 1935-1936 under the coordination of Gian Alberto Blanc [11].

During the 1970s-1980s, there was a notable increase in improved systematic research, leading to the discovery of several sites in the north-west sector of Rome Capanna Murata, Castel di Guido, La Polledrara di Cecanibbio and Torre del Pagliaccetto [12-18], along with Casal de’ Pazzi within the urban area of Rome [19]. The first four deposits are situated not far from Casal Lumbroso and provide evidence of the human presence and changes in terrestrial ecosystems between Marine Isotope Stages (MIS) 13 and 7, encompassing four different interglacials (i.e., MIS 13, 11, 9, and 7) of the Middle Pleistocene.

Capanna Murata was systematically excavated between 1975 and 1978, by the Institute of Anthropology and Human Paleontology of the University of Pisa [13,20]. Cassoli et al. [13] attributed lithic and bone tools to Acheulean documenting the human presence at the site. Additionally, they listed several mammal species found in the deposits (Table S1).

Castel di Guido also plays an important role for studying the human strategies behavioural aspects of the pre-Neandertal groups during MIS 11[21-23]. The site preserves intentionally fragmented bones of large mammals and Acheulean biface-like industry, including bifaces made of various stone types and of elephant bone [21-23]. Boschian et al.[23] demonstrated extensive exploitation of elephant long bones for marrow extractions, in addition to their use as raw material for tools. The mammal assemblage was comprehensively described by Sala & Barbi[24] (Table S1). The pre-Neanderthal specimens at the site include five cranial remains and two long bones [25-26].

La Polledrara di Cecanibbio, stands out as the most significant among these sites, providing a valuable glimpse into terrestrial ecosystems in Mediterranean Europe during the MIS 9. The deposit preserves thousands of bones, including at least three almost complete carcasses of *Palaeoloxodon antiquus* in anatomical connections, still trapped in mud sediments, immortalizing their death [18,27-29] (Table S1). The archeological study has also indicated that these carcasses were exploited by humans [18.27,30,31], especially the discovery of 600 lithic artifacts associated with one of the elephant carcasses [32]. Additionally, an isolated tooth attributed to a juvenile individual of *Homo heidelbergensis* aged between 5 and 10 years, was found in the deposit [33].

The last site, Torre del Pagliaccetto, is characterized by two distinct levels chronologically referred respectively to MIS 9 and MIS 7 [34 and references therein]. Systematic excavations were conducted during the 1950s and 1960s, coordinated by the Italian Institute of Human Paleontology [35]. The results of these excavations were reported in a series of works published on Quaternaria in 1978, gathering the geological, stratigraphic, paleontological, and archeological information. The lithic tools of the lower levels (n and m) were referred to Acheulean, whereas the sample collected in level d was attributed to the Middle Palaeolithic [34 and references therein]. Bone tools were also found in the level m [36]^.^ The faunal assemblages from the lower and upper levels were described by Caloi & Palombo [37] (Table S1).

Other deposits have been found near the Aurelia State Road, with their chronology dating back to the Middle Pleistocene, spanning between MIS 13 and MIS 11 [e.g., 38-39]. These include Capanna Murata [13], Malagrotta (Pio Istituto di Santa Spirito) [40], Collina Barbattini, Via Aurelia 19.3 Km, via Aurelia 19.0 Km, and Via Aurelia 18.9 Km [38-40] (Table S1). These sites commonly preserve mammal fossil remains as well as lithic and bone tools.

In this broad context, Casal Lumbroso represents one of the few systematically investigated sites in the Rome area dating back to the Middle Pleistocene. Furthermore, our results confirm that the north-west territory of Rome was frequented by pre-Neanderthals, who often exploited the carcasses of naturally dead elephants.

**References**

1. Palombo MR. Biochronology of Plio-Pleistocene mammalian faunas on the Italian Peninsula: Knowledge, problems and perspectives. Alp. Mediterr. Quat. 2004; 17: 565–582.

2. Kotsakis T, Barisone G. Cenni sui vertebrati fossili di Roma. Memorie Descrittive della Carta Geologica d'Italia. 2008; 80 (1): 115–143.

3. Romano M, Mecozzi B, Sardella R. The Quaternary paleontological research in the Campagna Romana (central Italy) at the 19th-20th century transition. Historical overview. Alp. Mediterr. Quat. 2021; 34 (1): 109–130. <https://doi.org/10.26382/AMQ.2021.06>

4. Iannucci A, Mecozzi B, Sardella R. Beware of the "Wolf event" - Remarks on large mammal dispersals in Europe and the late Villafranchian faunal turnover. Alp. Mediterr. Quat. 2023; 36 (1): 75–90. <https://doi.org/10.26382/AMQ.2023.03>

5. Gliozzi E, Abbazzi L, Argenti P, Azzaroli A, Caloi L, Capasso Barbato L, et al. Biochronology of selected mammals, molluscs and ostracods from the Middle Pliocene to the Late Pleistocene in Italy. Riv. It. Paleontol. Strat. 1997; 103 (3): 369–388. <https://iris.uniroma1.it/handle/11573/423099>

6. Petronio C, Sardella R. Biochronology of Plio-Pleistocene mammalian faunas on the Italian Peninsula: Knowledge, problems and perspectives. Riv. It. Paleontol. Strat. 1999; 105 (1): 155–164. <https://doi.org/10.13130/2039-4942/5370>

7. Milli S, Palombo MR, Petronio C, Sardella R. The Middle Pleistocene deposits of the Roman basin (Latium, Italy): An integrated approach of mammal biochronology and sequence stratigraphy. Riv. It. Paleontol. Strat. 2004; 110: 557–567.

8. Palombo MR, Filippi ML, Iacumin P, Longinelli A, Barbieri M, Maras A. Coupling tooth microwear and stable isotope analyses for palaeodiet reconstruction: The case study of Late Middle Pleistocene *Elephas* (*Palaeoloxodon*) *antiquus* teeth from Central Italy (Rome area). Quat. Int. 2005; 126-128: 153–170. <https://doi.org/10.1016/j.quaint.2004.04.020>

9. Strani F, Bellucci L, Iannucci A, Iurino DA, Mecozzi B, Sardella R. Palaeoenvironments of the MIS 15 site of Cava di Breccia-Casal Selce 2 (central Italian Peninsula) and niche occupation of fossil ungulates during Middle Pleistocene interglacials. Hist. Biol. 2022; 34 (3): 555–565. <https://doi.org/10.1080/08912963.2021.1935920>

10. Mecozzi B, Iannucci A, Mancini M, Sardella R. Redefining Ponte Molle (Rome, central Italy): An important locality for Middle Pleistocene mammal assemblages of Europe. Alp. Mediterr. Quat. 2021; 34 (1): 131–154. <https://doi.org/10.26382/AMQ.2021.09>

11. Blanc AC. Notizie sui ritrovamenti e sul giacimento di Saccopastore e sulla sua posizione nel Pleistocene laziale. Palaeontogr. Ital. 1948; 42: 1–24.

12. Longo E, Pitti C, Radmilli A. Prima campagna di Scavo nella stazione del Paleolitico inferiore a Castel di Guido presso Roma. Atti Soc. Tosc. Sci. Nat. Pisa. 1980; 87: 443–449.

13. Cassoli P, De Giuli C, Radmilli A, Segre A. Giacimento del paleolitico inferiore a Malagrotta (Roma). Atti della XXIII Riunione Scientifica dell'Istituto Italiano di Preistoria e Protostoria. 1982; 23: 531–49.

14. Anzidei AP, Sebastiani R. Saggi di scavo nel deposito pleistocenico al km 19,300 della Via Aurelia (Castel di Guido). Preistoria e Protostoria nel territorio di Roma. 1984; 3: 86–93.

15. Anzidei AP, Biddittu I, Cassoli PF, Segre AG. Le gisement pléistocène de la Polledrara di Cecanibbio (Rome, Italie). L'Anthropologie (Paris). 1989; 93(4): 749–81.

16. Radmilli AM. Un insediamento dell'*Homo erectus* a Castel di Guido presso Roma. Paleocronache. 1992; 2: 49–59.

17. Radmilli AM, Boschian G. Gli scavi a Castel di Guido: Il piu antico glacimento di cacciatori del paleolitico inferire nell'Agro Romano. Firenze: Istituto italiano di preistoria e protostoria; 1996.

18. Anzidei AP, Arnoldus-Huyzendveld A, Caloi L, Castorina F, Celant A, Cerilli E, et al. Ongoing research at the late Middle Pleistocene site of La Polledrara di Cecanibbio (central Italy), with emphasis on human-elephant relationships. Quat. Int. 2012; 255: 171–187. <https://doi.org/10.1016/j.quaint.2011.06.005>

19. Anzidei AP, Cassoli PF, Ruffo M, Segre AG. Risultati preliminari dello scavo in un deposito pleistocenico in località Rebibbia-Casal de' Pazzi. In: Atti del XXIV Convegno dell'Istituto Italiano di Preistoria e Protostoria. Roma; 1984. pp. 131–139.

20. Pennacchioni M, Persiani C. Presenze del Paleolitico inferiore nella zona di Malagrotta (Roma). Atti Istituto Italiano di Preistoria e Protostoria. 1982; 23: 551–553.

21. Boschian G, Saccà D. Ambiguities in human and elephant interactions? Stories of bones, sand and water from Castel di Guido (Italy). Quat. Int. 2010; 214 (1): 3–16. <https://doi.org/10.1016/j.quaint.2009.10.016>

22. Boschian G, Saccà D. In the elephant, everything is good: Carcass use and re-use at Castel di Guido (Italy). Quat. Int. 2015; 361: 288–96. <https://doi.org/10.1016/j.quaint.2014.04.030>

23. Boschian G, Caramella D, Saccà D, Barkai R. Are there marrow cavities in Pleistocene elephant limb bones, and was marrow available to early humans? New CT scan results from the site of Castel di Guido (Italy). Quat. Sci. Rev. 2019; 215: 86–97. <https://doi.org/10.1016/j.quascirev.2019.05.010>

24. Sala B, Barbi G. Descrizione della fauna. In: Radmilli AM, Boschian G, editors. Gli scavi a Castel di Guido, il più antico giacimento di cacciatori del Paleolitico inferiore nell'Agro Romano. Pisa. 1996; pp. 55–91.

25. Mallegni F, Mariani-Costantini R, Fornaciari G, Longo ET Giacobini G, Radmilli AM. New European fossil hominid material from an Acheulean site near Rome (Castel di Guido). Am. J. Phys. Anthropol. 1983; 62 (3): 263–74. <https://doi.org/10.1002/ajpa.1330620306>

26. Mallegni F, Radmilli AM. Human temporal bone from the Lower Paleolithic site of Castel di Guido, near Rome, Italy. Am. J. Phys. Anthropol. 1988; 76 (2): 175–82. <https://doi.org/10.1002/ajpa.1330760205>

27. Santucci E, Marano F, Cerilli E, Fiore I, Lemorini C, Palombo MR, et al. *Palaeoloxodon* exploitation at the Middle Pleistocene site of La Polledrara di Cecanibbio (Rome, Italy). Quat. Int. 2016; 406: 169–182. <https://doi.org/10.1016/j.quaint.2015.08.042>

28. Cerilli E, Fiore I, Santucci E, Marano F, Anzidei AP, Bulgarelli GM, et al. Strategie di sfruttamento delle risorse animali a La Polledrara di Cecanibbio (Pleistocene medio-superiore, Roma). Atti 8° Convegno Nazionale di Archeozoologia (Lecce, 2015). [University of Salento] 2015: pp. 21–30.

29. Cerilli E, Lemorini C, Santucci E, Fiore I, Marano F, Bulgarelli GM, et al. Environment and daily life in the Campagna Romana of the late Lower Palaeolithic: the case-study of La Polledrara di Cecanibbio (Latium, Italy). J. Mediterr. Earth Sci. 2023; 15: 91–108. <https://iris.uniroma1.it/handle/11573/1688496>

30. Anzidei AP, Arnoldus-Huyzenveld A, Caloi L, Lemorini C, Palombo MR. Two Middle Pleistocene sites near Rome (Italy): La Polledrara di Cecanibbio and Rebibbia-Casal de' Pazzi. In: Gaudzinski S, Turner E, editors. The Role of Early Humans in the Accumulation of European Lower and Middle Paleolithic Bone Assemblages. Mainz: Monographien des Römisch-Germanischen Zentralmuseums 42; 1999: pp. 173–95. <https://iris.uniroma1.it/handle/11573/90013>

31. Anzidei AP, Biddittu I, Cassoli PF, Segre AG. Nouvelles données sur le gisement pléistocène moyen de la Polledara di Cecanibbio. In: Miscelánea en Homenaje a Emiliano Aguirre, Arqueología 4. Museo Arqueológico Regional; 2004: pp. 20–9.

32. Lemorini C, Santucci E, Caricola I, Nucara A, Cesaro SN. Life Around the Elephant in Space and Time: An Integrated Approach to Study the Human-Elephant Interactions at the Late Lower Paleolithic Site of La Polledrara di Cecanibbio (Rome, Italy). J. Archaeol. Method Theory. 2023; 30 (4): 1233–1281. <https://doi.org/10.1007/s10816-022-09584-4>

33. Manzi G, Magri D, Palombo MR. Early-Middle Pleistocene environmental changes and human evolution in the Italian peninsula. Quat. Sci. Rev. 2011; 30 (11): 1420–1438. <https://doi.org/10.1016/j.quascirev.2010.05.034>

34. Villa P, Soriano S, Grün R, Marra F, Nomade S, Pereira A, et al. The Acheulian and Early Middle Paleolithic in Latium (Italy): Stability and Innovation. PLOS ONE. 2016; 11 (8): e0160516. <https://doi.org/10.1371/journal.pone.0160516>

35. Malatesta A. La serie di Torre del Pagliaccetto e il bacino di Torre in Pietra. Quaternaria. 1978; 20: 237–246.

36. Piperno M, Biddittu I. Studio tipologico e interpretazione dell'industria acheuleana e premusteriana dei livelli m e d di Torre in Pietra (Roma). Quaternaria. 1978; 20: 441–536.

37. Caloi L, Palombo MR. Resti di mammiferi del Pleistocene Medio di Malagrotta (Roma). Boll. Serv. Geol. It. 1979; 100: 141–188.

38. Ceruleo P, Rolfo MF, Petronio C, Salari L. Review of Middle Pleistocene archaeological and biochronological data in Malagrotta-Ponte Galeria area (Rome, Central Italy). Atti Soc. Tosc. Sci. Nat., Memorie, Serie A. 2021; 128: 85–103.

39. Anzidei AP, Biddittu I, Cassoli PF, Segre AG. Saggi di scavo nei depositi pleistocenici del KM 18,900 della Via Aurelia e di Collina Barbattini (Castel di Guido-Roma). *Archeologia Laziale XI: Undicesimo incontro di studio del Comitato per l'Archeologia Laziale (Estratto). Quaderni di Archeologia Etrusco-Italica. 1983; 21. <https://iris.uniroma1.it/handle/11573/396166>

40. Marra F, Nomade S, Pereira A, Petronio C, Salari L, Sottili G, et al. A review of the geologic sections and the faunal assemblages of Aurelian Mammal Age of Latium (Italy) in the light of a new chronostratigraphic framework. Quat. Sci. Rev. 2018; 181: 173–199.

[https.//doi.org/10.1016/j.[quascirev.2017.12.007](https://doi.org/10.1016/j.quascirev.2017.12.007)](https://doi.org/10.1016/j.quascirev.2017.12.007)

41 Venditti F, Rodríguez-Álvarez B, Serangeli J, Cesaro SN, Walter R, Conard N, et al. Using microartifacts to infer Middle Pleistocene lifeways at Schöningen, Germany. Sci. Rep. 2022; 12 (1): 21148. <https://doi.org/10.1038/s41598-022-24769-3>

42. Caloi L, Palombo MR. Resti di mammiferi del Pleistocene Medio di Malagrotta (Roma). Boll. Serv. Geol. It. 1979; 100: 141–188.

43. Iannucci A, Mecozzi B, Sardella R. Large mammals from the Middle Pleistocene (MIS 11) site of Fontignano 2 (Rome, central Italy), with an overview of "San Cosimato" assemblages. Alp. Mediterr. Quat. 2021; 34: 155–164.

44. Sydney-Zax M, Mayer I, Deutsch D. Carbonate content in developing human and bovine enamel. J. Dent. Res. 1991; 70: 913–916.
