# Supplementary figures and images for: From meat to raw material: the Middle Pleistocene elephant butchery site of Casal Lumbroso (Rome, central Italy)
Source: PLoS One. 2025 Oct 8;20(10):e0328840. doi: 10.1371/journal.pone.0328840 (PMC12507280; doi:10.1371/journal.pone.0328840)

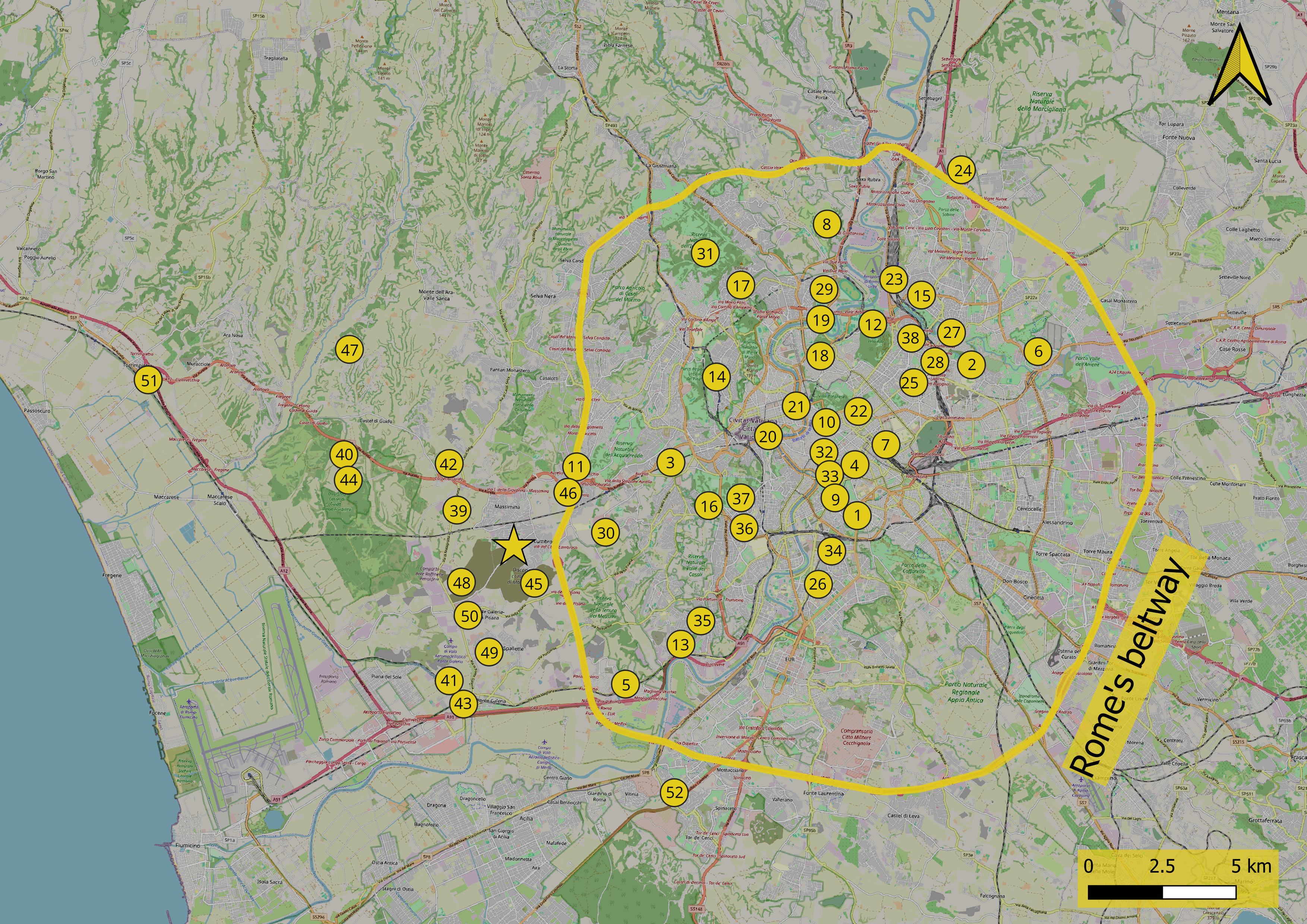

Supplement: S1 Fig — 1 – Aventino; 2 – Batteria Nomentana; 3 – Boccea; 4 – Campidoglio; 5 – Campo di Merlo; 6 – Casal de’ Pazzi, Ponte Mammolo; 7 – Castro Pretorio; 8 – Cava Nera Molinario; 9 – Monte Celio; 10 -Fondamenta BNL, Pincio, Quirinale; 11 – GRA 2 Km; 12 – Monte Antenne; 13 – Monte delle Pliche; 14 – Monte Mario; 15 – Monte Sacro; 16 – Monte Verde; 17 – Monti della Farnesina; 18 – Parioli; 19 – Ponte Molle; 20 – Porta Cavalleggeri; 21 – Porta Flaminia; 22 – Porta Pia, Porta Salaria; 23 – Prati Fiscali; 24 – Redicicoli; 25 – Sant’Agnese; 26 – San Paolo; 27 – Saccopastore; 28 – Sedia del Diavolo; 29 – Tor di Quinto, Cava Montanari; 30 – Via Aurelia; 31 – Via Cassia; 32 – Via del Tritone; 33 – Via Nazionale; 34 – Via Ostiense; 35 – Via Portuense; 36 – Vigna San Carlo; 37 – Vigne Torte; 38 – Villa Chigi; 39 – Capanna Murata, Pio Istituto di Santo Spirito; 40 – Castel di Guido; 41 – Cava Arnolfi, Cava Alibrandi, Muratella di Mezzo; 42 – Cava di Breccia di Casal Selce; 43 – Cava Rinaldi; 44 – Collina Barbattini, Via Aurelia 18.9 Km, 19.0 Km and 19.3 Km; 45 – Fontignano; 46 – La Maglianella; 47 – La Polledrara di Cecanibbio; 48 – Pantano di Grano; 49 – San Cosimato – Santa Cecilia; 50 – Via della Pisana; 51 – Torre del Pagliaccetto; 52 – Vitinia. Yellow star – Casal Lumbroso. (PNG) [file pone.0328840.s002.png]

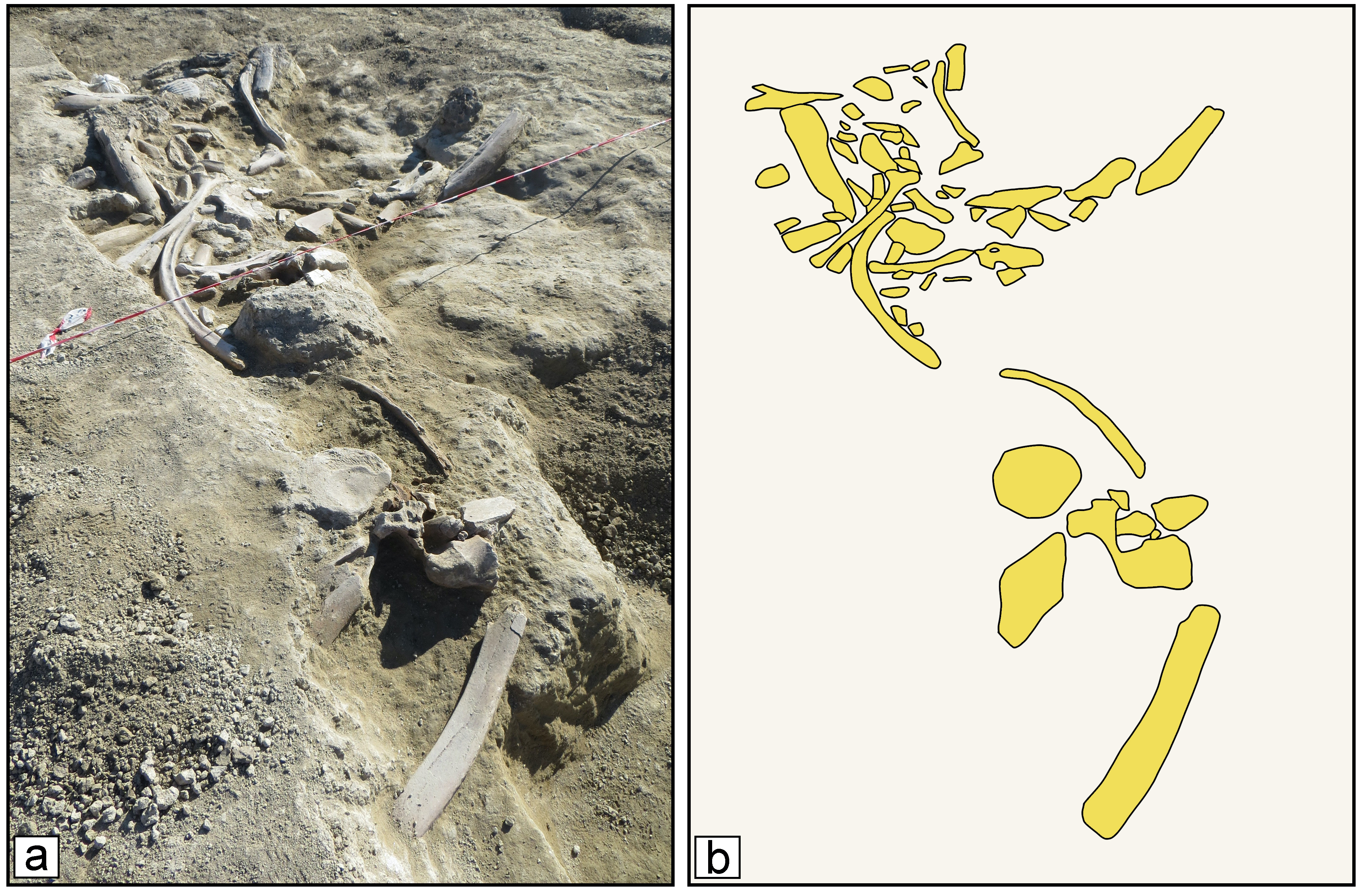

Supplement: S2 Fig — (JPG) [file pone.0328840.s003.jpg]

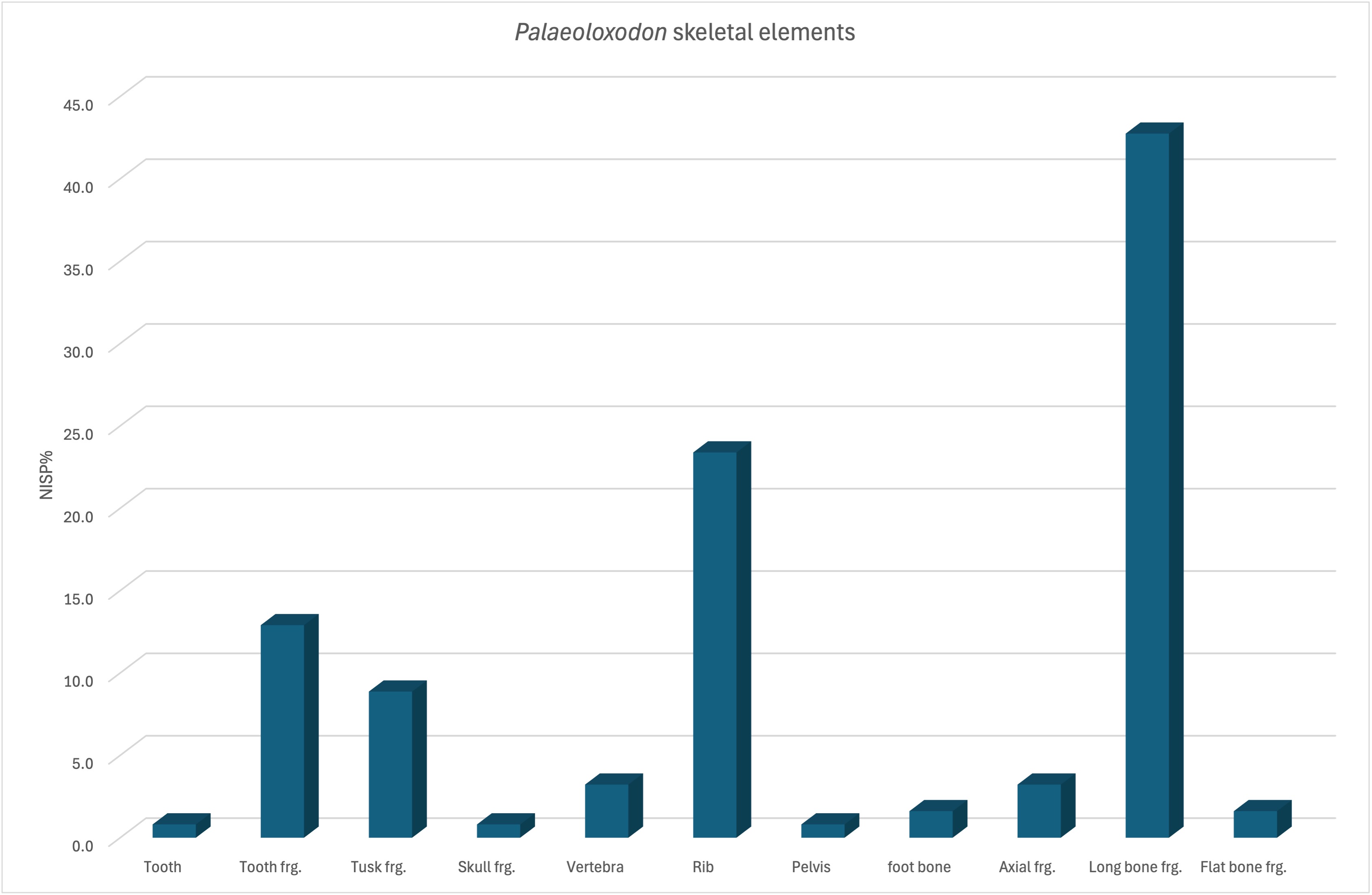

Supplement: S3 Fig — (JPG) [file pone.0328840.s004.jpg]

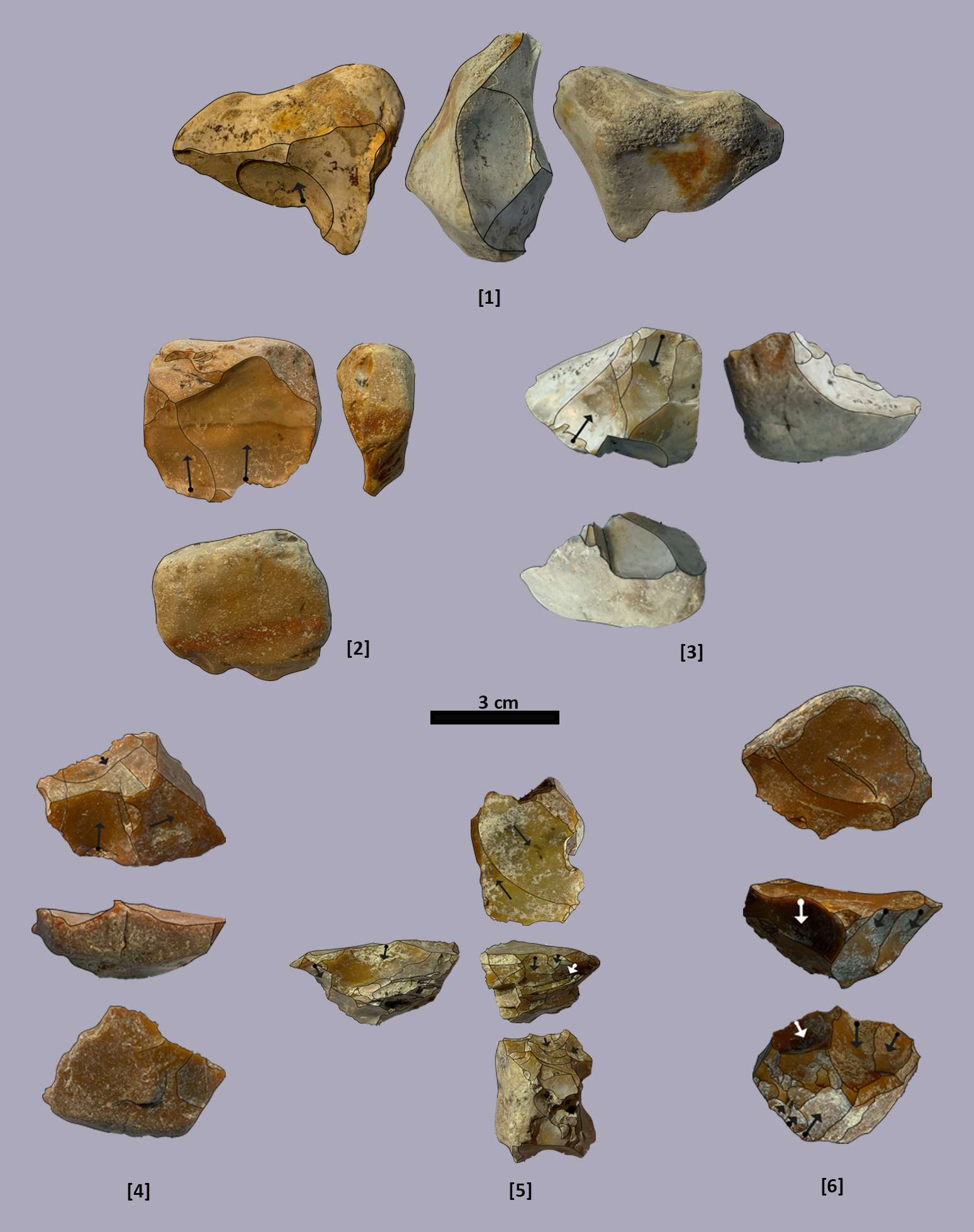

Supplement: S4 Fig — Unidirectional recurrent (1,2), bidirectional recurrent (3), unidirectional on core on flake (4), centripetal on core on flake (5) and centripetal (6). (JPG) [file pone.0328840.s005.jpg]

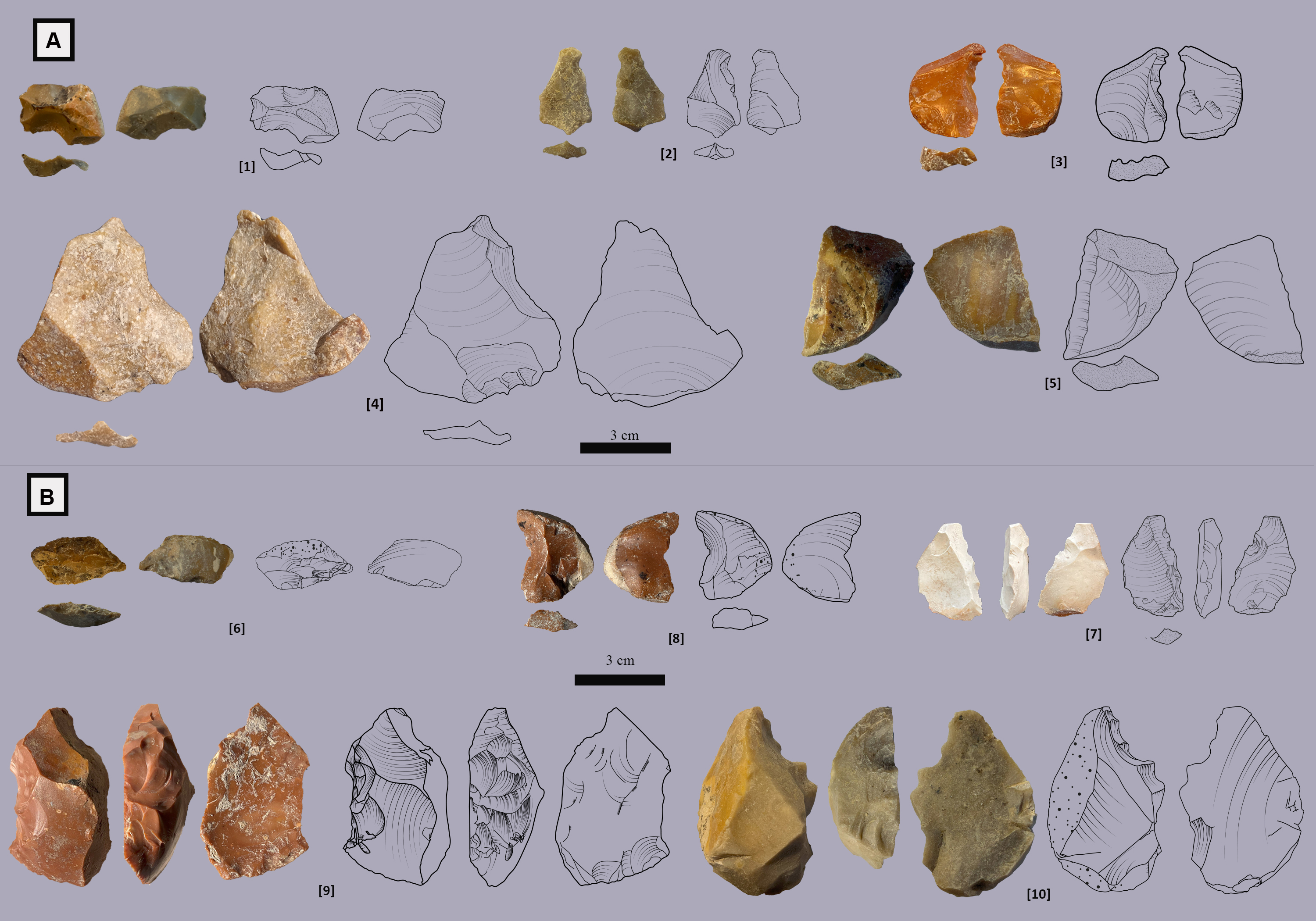

Supplement: S5 Fig — Flint flakes: centripetal flakes (1,2), convergent flakes (3,8), bidirectional irregular (5,6), and unidirectional (7); Chert flakes: centripetal flake (4); Limestone flakes: cortical (9), unidirectional (10,11[refitting flakes]). (JPG) [file pone.0328840.s006.jpg]
