# Supplementary material for: From meat to raw material: the Middle Pleistocene elephant butchery site of Casal Lumbroso (Rome, central Italy)
Source: PLoS One. 2025 Oct 8;20(10):e0328840. doi: 10.1371/journal.pone.0328840 (PMC12507280; doi:10.1371/journal.pone.0328840)
Supplement: S2 Table — To evaluate the possibility of contamination from external carbonate sources, we assessed the carbonate content (CaCO3%) in the enamel. The range of CaCO3 in the enamel structural carbonates, from 4.7 to 5.8%, falls within the range of CaCO3 contents of modern enamel (~3.0 − 5.5%) (58) and ungulate enamel bioapatite (4.5 − 5.1%) (59). (DOCX) [file pone.0328840.s008.docx]

| **Sample** | **Distance from the occlusal surface (mm)** | **δ^13^C**  **(‰ VPDB)** | **δ^18^O**  **(‰ VPDB)** | **δ^18^O**  **(‰ VSMOW)** | **CaCO_3_ (%)** |
| --- | --- | --- | --- | --- | --- |
|  |  |  |  |  |  |
| CL59A | 22 | −13.15 | −7.40 | +23.28 | 5.3 |
| CL59B | 25 | −13.15 | −6.78 | +23.92 | 4.9 |
| CL59C | 28 | −13.36 | −7.25 | +23.44 | 4.7 |
| CL59D | 31 | −13.03 | −7.12 | +23.57 | 5.3 |
| CL59E | 34 | −12.72 | −6.02 | +24.71 | 5.0 |
| CL59F | 37 | −12.51 | −6.91 | +23.79 | 5.4 |
| CL59G | 40 | −12.15 | −5.81 | +24.92 | 5.2 |
| CL59H | 43 | −13.05 | −6.83 | +23.86 | 4.7 |
| CL59I | 46 | −12.93 | −6.46 | +24.25 | 5.2 |
| CL59L | 49 | −12.79 | −6.98 | +23.72 | 5.2 |
| CL59M | 52 | −12.80 | −6.56 | +24.15 | 5.0 |
| CL59N | 55 | −12.60 | −7.05 | +23.64 | 5.2 |
| CL59O | 58 | −12.76 | −6.52 | +24.19 | 5.0 |
| CL59P | 61 | −12.79 | −6.94 | +23.76 | 5.5 |
| CL59Q | 64 | −12.56 | −6.97 | +23.72 | 5.8 |
| CL59R | 67 | −12.81 | −7.19 | +23.50 | 5.6 |
|  |  |  |  |  |  |
| **Mean** |  | −12.82 | −6.80 | +23.90 | 5.19 |
| **Standard deviation** |  | 0.29 | 0.43 | 0.45 | 0.30 |
